# Local Gene Regulation Details a Recognition Code within the LacI Transcriptional Factor Family

**DOI:** 10.1371/journal.pcbi.1000989

**Published:** 2010-11-11

**Authors:** Francisco M. Camas, Eric J. Alm, Juan F. Poyatos

**Affiliations:** 1 Logic of Genomic Systems Laboratory, Spanish National Biotechnology Centre, Consejo Superior de Investigaciones Científicas (CSIC), Madrid, Spain; 2 Department of Biological Engineering, Massachusetts Institute of Technology (MIT), Cambridge, Massachusetts, United States of America; Washington University School of Medicine, United States of America

## Abstract

The specific binding of regulatory proteins to DNA sequences exhibits no clear patterns of association between amino acids (AAs) and nucleotides (NTs). This complexity of protein-DNA interactions raises the question of whether a simple set of wide-coverage recognition rules can ever be identified. Here, we analyzed this issue using the extensive LacI family of transcriptional factors (TFs). We searched for recognition patterns by introducing a new approach to phylogenetic footprinting, based on the pervasive presence of local regulation in prokaryotic transcriptional networks. We identified a set of specificity correlations –determined by two AAs of the TFs and two NTs in the binding sites– that is conserved throughout a dominant subgroup within the family regardless of the evolutionary distance, and that act as a relatively consistent recognition code. The proposed rules are confirmed with data of previous experimental studies and by events of convergent evolution in the phylogenetic tree. The presence of a code emphasizes the stable structural context of the LacI family, while defining a precise blueprint to reprogram TF specificity with many practical applications.

## Author Summary

Transcriptional factors (TF) are proteins that bind specific short DNA sequences adjacent to the genes whose transcription they regulate. Although the nucleotide sequence recognized by a given regulator depends on the amino acids contacting the DNA, the mode in which amino acids and nucleotides interact is strongly influenced by the overall protein structure. This prevents the existence of a universal amino acid/nucleotide recognition code. However, recognition rules could be formulated for regulators sharing a similar structure, i.e., for a family or subfamily of TFs. In fact, such rules have already been described for several sets which, in each case, involved a limited number of related TFs. In this study, we ask to what extent a wide-coverage recognition code might actually be found. To answer this question, we use the extensive LacI family of transcriptional regulators. Our analysis suggests that a set of relatively consistent recognition rules does apply within a major subset of this family. These rules could ultimately act as a blueprint for the synthetic redesign of TFs with new specificities.

## Introduction

The search for principles describing how specific nucleotide sequences are recognized by proteins remains one of the most fundamental problems to be solved in Biology [1]–[5]. The relevance of this question is linked to the wide breadth of basic cellular processes to be better understood with its resolution, like how genomes respond to stress by accurately activating/inactivating groups of genes, or how cells differentiate into separate classes following a program of precise spatio-temporal gene expression. Additionally, these principles could turn into genuine rules to engineer protein production, either in isolation or as part of elaborated molecular circuits or networks, with many practical applications.

Given the relevance of this search, when could one say that principles have been actually identified, or that this goal failed? Answers to these questions changed over the years, e.g., [2], [6]–[10], as the knowledge of how transcriptional factors (TFs) recognize their cognate binding sites (BSs) did. Two mechanistic aspects of this recognition are relevant in this regard [11]–[13], i.e., how selected AA/NT binding partners determine specificity (direct readout) and how specificity could be influenced by additional structural features (indirect readout). Within this second aspect, both the protein structural context in which the contacting AAs are embedded [7], [14], [15] and the conformational characteristics of DNA upon TF binding [11], [12] appear as particularly important modifiers.

In fact, the relative strength of direct and indirect readouts can greatly influence the nature of the recognition rules to be identified. The most simplistic situation could be one in which (simple) direct readouts for the contacting positions were dominant specificity determinants. In this case, one could conceive the presence of deterministic codes of wide applicability. However, the rich repertoire for AA/NT interactions, which includes hydrogen or water-mediated bonds and also van der Waals contacts [16], and the context dependence of these interactions rule out the appearance of deterministic codes [6], [7], [17]. Instead, one should rather look for probabilistic recognition codes restricted to similar protein structures [3], [8], [14], [18]. The applicability of these principles to large protein groups might ultimately depend on the conservation of the modifiers linked to indirect readouts.

Interestingly, some of these issues can be studied with the use of mutational experiments –either *in vivo* [19], [20] or *in vitro* [8], [10], [21], [22] – which start with a known TF/BS relationship to characterize changes in specificity once selected AA and/or NT positions are mutated. Since the number of possible sequences grows exponentially with the number of positions to be explored, this approach usually requires the use of large mutant libraries. Consequently, even when the sequence space is explored in a random way [8], or by screening methods [20], the positions to be mutated are always selected among those corresponding to direct readouts. Since the rest of positions remains fixed, the conservation of the structural context within the library directly follows. This implies that any set of recognition rules deduced from the mutational approach is restricted, in principle, to the library elements.

The existence of a natural version of such synthetic code would require a strong conservation of the mode of binding within the family of proteins to which the focal mutated protein belongs –despite the variability in the non-contacting positions [23], [24]. Mutational studies can estimate this conservation only in an indirect manner, by finding natural correspondences of some of the synthetic AA/NT relationships studied [8], [19]. Regardless of the existence or absence of such correspondences, those mutants with differential specificities could constitute useful tools for Synthetic Biology [20], [25].

An alternative approach to this problem, in which the role of indirect readouts is evaluated, deduces the recognition rules by using genomic tools applied to natural sequences of both TFs and BSs [14], [15], [26]–[28]. In this case, each residue/base contact is embedded in its own structural context and the possibility of family codes can be explicitly examined. The finding of consistent recognition rules, whereby the sequences of the contacting AAs and NTs correlate, would imply that variations on the rest of residues do not compromise the conservation of the binding mode within the considered set. Moreover, such natural recognition code would suggest that the evolution of new specificities is mainly achieved by alteration of base contacting residues (direct readouts) [14]. Recognition rules following this approach were formulated for several sets which, in each case, involved a limited number of related TFs [14], [27], [28].

In this work, we asked to what extent a natural wide-coverage recognition code could exist. From the arguments before, this code could be considered as such when it fulfills two important requirements. First, the determinants of the indirect readout should not prevent the identification of consistent sequence correlations between the contacting AAs and NTs for a given regulator family (or a substantial fraction of it). Second, most of these natural associations should be reproducible by mutating the specificity-associated AAs of a particular focal member of the family. Note that these features do not include that the recognition correlations should be expressed in terms of a few deterministic rules –although strong general trends are expected.

**Figure 1:**
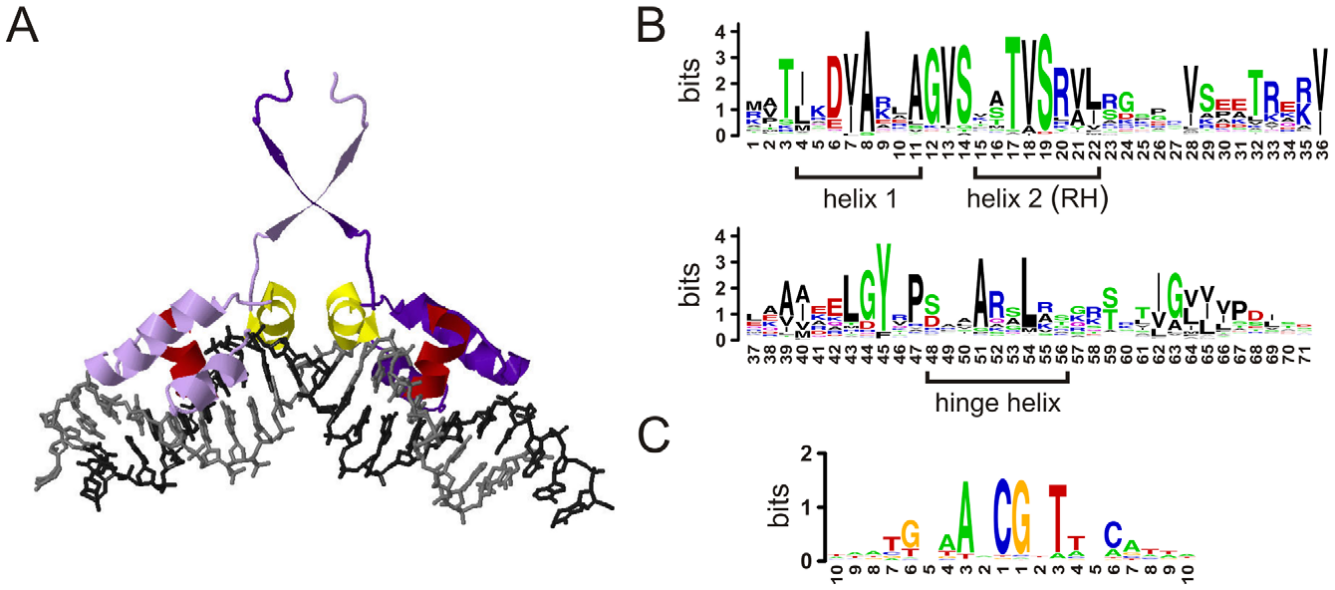
HTH binding mode. A) X-ray model for a LacI dimer bound to a palindromic BS (plotted with Jmol from the PDB structure 1lbg4). Only the binding domain of each monomer is shown (in light/dark purple, respectively). The hinge-helix and the recognition helix of each monomer are colored in yellow and red, respectively. B) Logo for the alignment of 2639 unredundant HTH-LacI domains. The AA coordinates of any particular domain will be referred by its position in this alignment –they match the numbering of the first 71 AAs of *Escherichia coli*'s GalR and GalS regulators. Helix-1, helix-2 (or recognition helix) and the intermediate residues constitute the HTH motif itself. C) Logo for the alignment of the set of BSs associated to 370 LacI family members (BS sequences from RegTransBase [46]). In BS logos we avoided subscripts for left and right half sites coordinates.

We considered as a model system to approach this question the extensive LacI family of transcriptional regulators [26], whose helix-turn-helix (HTH) domain (Fig. 1.A) interacts with a set of cognate BSs [29]. Within this set, we examined a dominant group (involving more than half of the LacI family members) composed by regulators exhibiting the sequence threonine-valine-serine-arginine (TVSR) in the recognition helix of the HTH domain. We searched for recognition rules by introducing a new strategy based on comparative genomics and the use of a pervasive characteristic of prokaryotic regulation: the local control of gene expression [30]–[34].

Our analysis suggests that the determinants of the indirect readout are substantially conserved throughout the TVSR group, in which a set of relatively consistent recognition rules applies. Moreover, the phylogenetic tree associated to this group exhibited several convergence events for the recognition relationships, i.e., distant proteins in the tree sharing the same recognition AA sequence tend to bind similar NT sequences. The natural recognition correlations identified could be reproduced with a synthetic approach, as suggested by comparing the theoretical predictions with previous mutational experiments [19], [20] and by the finding of natural BSs previously considered as simple laboratory constructs [35].

## Results/Discussion

### Same binding patterns could be pervasive to the whole LacI family

We aligned non-redundant HTH-LacI domain sequences using information from MicrobesOnline [36], a database that contains approximately one thousand prokaryotic genomes (Methods). The resulting sequence logo (Fig. 1.B) suggested that the binding patterns previously identified with structural studies could potentially apply to the whole LacI family. Specifically, these studies solved the binding-domain/DNA complex of *Escherichia coli*'s LacI [37]–[39] and PurR [40], [41], and *Bacillus megaterium*'s CcpA [42], clearly distinguishing a contrast between structural and DNA-binding residues in the corresponding domains.

Indeed, positions exhibiting a strong conservation in our comparative analysis corresponded to proposed structural residues. In particular, the conservation of the hydrophobic residues in AA-54 (mostly leucine, 82%) indicated that the BS pattern in the family could be dominated by a conserved central CG group (although we did not use this prior knowledge in our analysis). In every structural study, this residue of the hinge-helix inserts into a central CG group located in the minor groove and bends the DNA (Fig. 1.A). The conserved alanine in AA-51 is similarly related in these analyses to the hinge-helix/CG union by non-specific interactions with the phosphate groups, or through direct contacts with the bases [43]. Exceptions to this union are rare [44], [45].

To identify the potential DNA-binding residues resolving BS specificity, we selected those domains in the alignment which were univocally associated to BSs in the RegTransBase v5 [46] (370 domains). These BSs were aligned to produce the logo in Fig. 1.C. Note the palindromic nature of this logo, which manifests the symmetrical contacts made by the monomers that constitute the dimeric regulators on the corresponding left(*L*)/right(*R*) half site location of the BSs [in the following, we usually simplify the notation of symmetrical positions, and palindromic sequences, by those in the left half site, e.g., 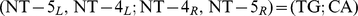 as (NT-5, NT-4) = TG].

We then calculated the mutual information (covariance dependency) between the alignment of these 370 domains and that of their corresponding BSs [47] (Fig. S1). This computation identified three main patterns. First, the extensive linkage between the non-conserved nucleotide pair (NT-5, NT-4) and the (AA-15, AA-16) residues located in the recognition helix (this helix includes residues AA-15 to AA-22, see Fig. 1.B). Second, the presence of a strong connection between NT-6 and AA-20 (also in the recognition helix); these coordinates exhibited no other appreciable interdependences suggesting a mode of recognition relatively independent to the previously discussed pair. Finally, the correlation of NT-2 with AA-55, AA-15 and AA-5, in decreasing order of importance.

The mutual information analysis also generalized previous experimental results obtained with a few members of the LacI family, this time with respect to the proposed specificity residues. In particular, the association of the pair (NT-5, NT-4) to (AA-15, AA-16) was demonstrated by structural models [29] and mutational studies [19]. The independent nature of the recognition interaction between NT-6 and AA-20 was also suggested by previous mutational studies of *E. coli*'s LacI [19], [29]. In addition, the link between NT-2 and the hinge-helix residue AA-55 (Fig. 1.B) was proposed in [41]. Moreover, although AA-20 was related to recognition processes, it is a strongly conserved residue –with arginine (R) linked to the presence of a guanine in NT-6 (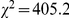, 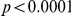, Yates-corrected 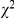-test). This resulted in the same AA sequence (a TVSR sequence for the range AA-17 to AA-20) in 1490 instances of a total of 2639 included domains (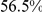, Fig. S2). We thus restricted the following analysis to the TVSR dominant subgroup.

From all the above, we hypothesized that the distinction among the different BSs associated to the TVSR set would rely mostly on the (AA-15, AA-16) pair. We further considered a stronger version of this hypothesis assuming that regulators sharing the same (AA-15, AA-16) sequence would tend to bind similar BSs regardless of their evolutionary distance. In the following, we tried to confirm these conjectures by analyzing the possible presence of a recognition code assigning specific nucleotides (NT-5, NT-4) to residues (AA-15, AA-16).

### Autoregulation helps identify a recognition code

The search of a wide-coverage recognition code required a large scale identification of the native BSs for each TF, with independence of its location in the LacI family phylogenetic tree. This requirement might become problematic if we were to apply the standard protocols of BS search. These methods often rely on the identification of orthologs of experimentally determined target genes to look for conserved upstream BSs –for example, by applying phylogenetic footprinting (PF) techniques [48]. As evolutionary distance between TFs increases, this approach lacks precision because of the complications to define orthologs, e.g., due to events of duplication and loss of genes [49].

**Figure 2:**
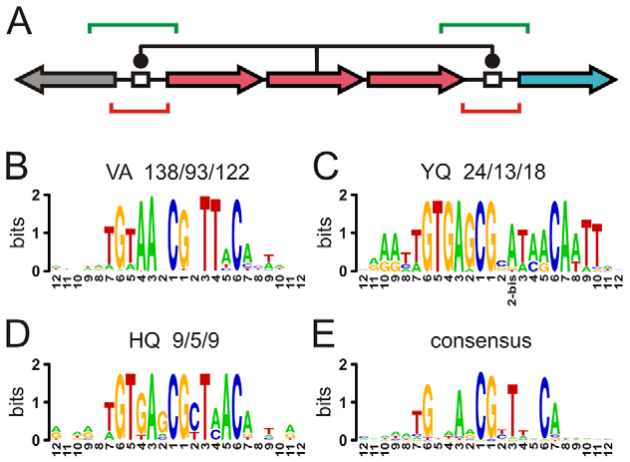
Autoregulation and the search of conserved binding sequences. A) Local regulation at the core of phylogenetic footprinting includes both autoregulation –which can be linked to the regulation of an upstream divergent operon– and downstream unidirectional adjacent regulation (BSs, white boxes). Red and green lines for the respective strict and extended regions of BS search. B–D) Examples of BS logos. Rest of cases in the Appendix of Text S1. Above each logo: the recognition sequence (AA-15, AA-16) and a triad of numbers (i/ii/iii) corresponding to i) the total number of TFs exhibiting the recognition sequence, ii) the number of TFs for which at least a BS was found, and iii) the total number of found BSs. E) Consensus-logo for the BSs associated to the TVSR group. The inserted position NT-2bis for the YQ-logo in C) has not been considered to build the consensus.

We decided to use a complementary strategy to search for BSs. This strategy was based on the hypothesis of the conservation of binding mode and also on the widespread presence of local transcriptional control in bacteria (including both auto- and neighbor-regulation [34]). Thus, we first grouped regulators sharing the same sequence of recognition residues (AA-15, AA-16), regardless of the evolutionary distance among the full TF sequences. Within each of these groups, or recognition classes, we looked for potential BSs in the intergenic regions located before the operon encoding the TF itself, and before the downstream operon, respectively (Fig. 2.A). We applied PF for BS search on these sequences with a subsequent refinement based on iterated position weight matrices (PWMs) (this protocol was aimed to minimize the rate of false positives linked to bioinformatic BS searches [49], see Methods).

We obtained in this way a nucleotide logo from each alignment of BSs associated to a recognition class (Figs. 2.B–D and Appendix in Text S1 for the complete set). We also computed the consensus logo of the full TVSR group (Fig. 2.E), where the contrast between conserved and non-conserved NTs is especially apparent. Although we used uninformed priors in the BS-finding algorithms to avoid circularity biases, the obtained consensus logo corresponded to the one expected from a situation where the TF binding mode is conserved (compare Fig. 1.C, computed from a previously known BS set [46], to Fig. 2.E). Note the conservation of G in 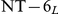 (and C in 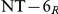), for we considered a group of domains with arginine in AA-20. Computation of the familial binding profile [23], [24] –a method that can also suggest the conservation of the binding mode within a TF family– for the TVSR set produced the same qualitative patterns in the consensus logo.

### Two contrasting scenarios to test for a wide-coverage recognition code

Once we obtained the BS logos associated to each AA recognition class, we could naively suppose that the presence of logos with high information content in both NT-4 and NT-5 would confirm the hypothesis of a recognition code. In the same vein, ambiguities in these nucleotides would reject the hypothesis (for example, in the set 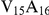, Fig. 2.B, where both T and A are found in 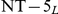). However, low-information positions could alternatively be explained by degeneracies in the recognition process, an expected attribute of extant codes [3]. In this latter case, the code conjecture would still hold true.

How can we distinguish these contrasting situations? Imagine a simplistic scenario in which a particular recognition AA sequence corresponds to a (recognition) class uniquely constituted by two different TFs. Imagine also that there were only two types of half site with different (NT-5, NT-4) sequences in the BSs observed for this TF class. Consequently, the corresponding BS logo would exhibit low-information (NT-5, NT-4) positions. This ambiguity could be caused because the particular (AA-15, AA-16) sequence for this class showed some degeneracy in recognition (as discussed above; we termed this intrinsic degeneracy), or because each TF exhibited a precise specificity to either type of half site, i.e., the recognition AA pair is not acting as the only determinant of specificity.

**Figure 3:**
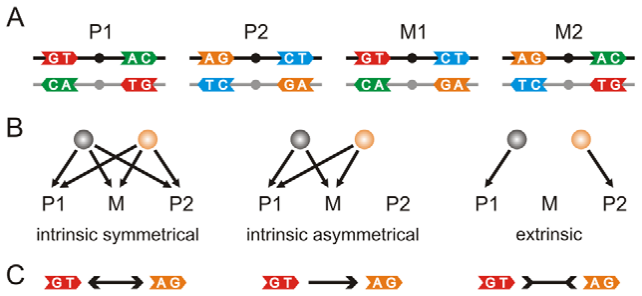
Degeneracies in TF binding. A) Palindromic (P1 and P2) and non-palindromic BSs (M1 and M2). Nucleotides (NTs) in positions 4 and 5 in both half sites and strands were only considered. Colors distinguished different NTs pairs. Only the sense strand (black line) is included in the alignment of BSs. Half sites separated by dots. B) Scenarios for degeneracy. Spheres represent two different TFs sharing the same recognition amino acids. Arrows indicate what BSs they can bind. We considered both mixtures to have the same binding energy and termed them simply as M. See main text for details. C) Notation criterion for degeneracies uses different arrows between the corresponding left semisequences 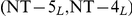 in the sense strand of the palindromic combinations.

We can further illustrate this with the help of Figure 3. In principle, the two species of half sites involved could be combined into palindromic (P1, P2 in Fig. 3.A) or non-palindromic architectures (M1, M2 in Fig. 3.A). When each TF monomer had a high affinity for both half sites (Fig. 3.B left), they could bind efficiently to P1, P2 and either mixture (we considered both mixtures to have the same binding energy). In a second situation (Fig. 3.B, center) both TFs had again similar affinities, but this time the monomers bound preferentially to one type of half site and, consequently, to one palindrome. Although a mixed configuration could still be compatible with (weaker) regulatory tasks, the probability of binding to the other palindrome strongly decreased. These are two instances of intrinsic degeneracy. Finally, in a third scenario each TF was very specific to a single half site type; so that only P1 or P2 were accessible (no mixtures), an example of logo ambiguity due to an extrinsic degeneracy (Fig. 3.B, right).

Ambiguities explained as intrinsic degeneracies are compatible with our starting hypothesis and would only reflect a degenerate code. The code hypothesis must be revised or even rejected when extrinsic degeneracies are common. This would presumably reflect critical changes in the determinants of the indirect readout.

### Comparative data suggests the presence of a wide-coverage code

A BS logo can thus be degenerate because i) the recognition process is degenerate in itself (intrinsic degeneracy) or ii) the logo is computed from BSs recognized by TFs with different specificities (extrinsic degeneracy). To distinguish between these two scenarios, we identified and classified degeneracies (Methods). Fig. 3.C shows the notation used for the different degeneracies. One could simultaneously observe several of these degenerate scenarios for any alignment involving more than two different types of half sites.

Table S1 included all correlations obtained between the pair of residues (AA-15, AA-16) and the nucleotides NT-4 and NT-5, together with the corresponding degeneracies when observed. This table contains 48 different recognition classes, involving a total of 38 intrinsic and 6 extrinsic degeneracies (some classes exhibiting both). The different types of identified degeneracies corroborated the potential of this protocol to detect distinct BSs within a TF class. The extrinsic degeneracies observed constitute a small number of exceptions to an otherwise consistent confirmation of the code conjecture.

**Figure 4:**
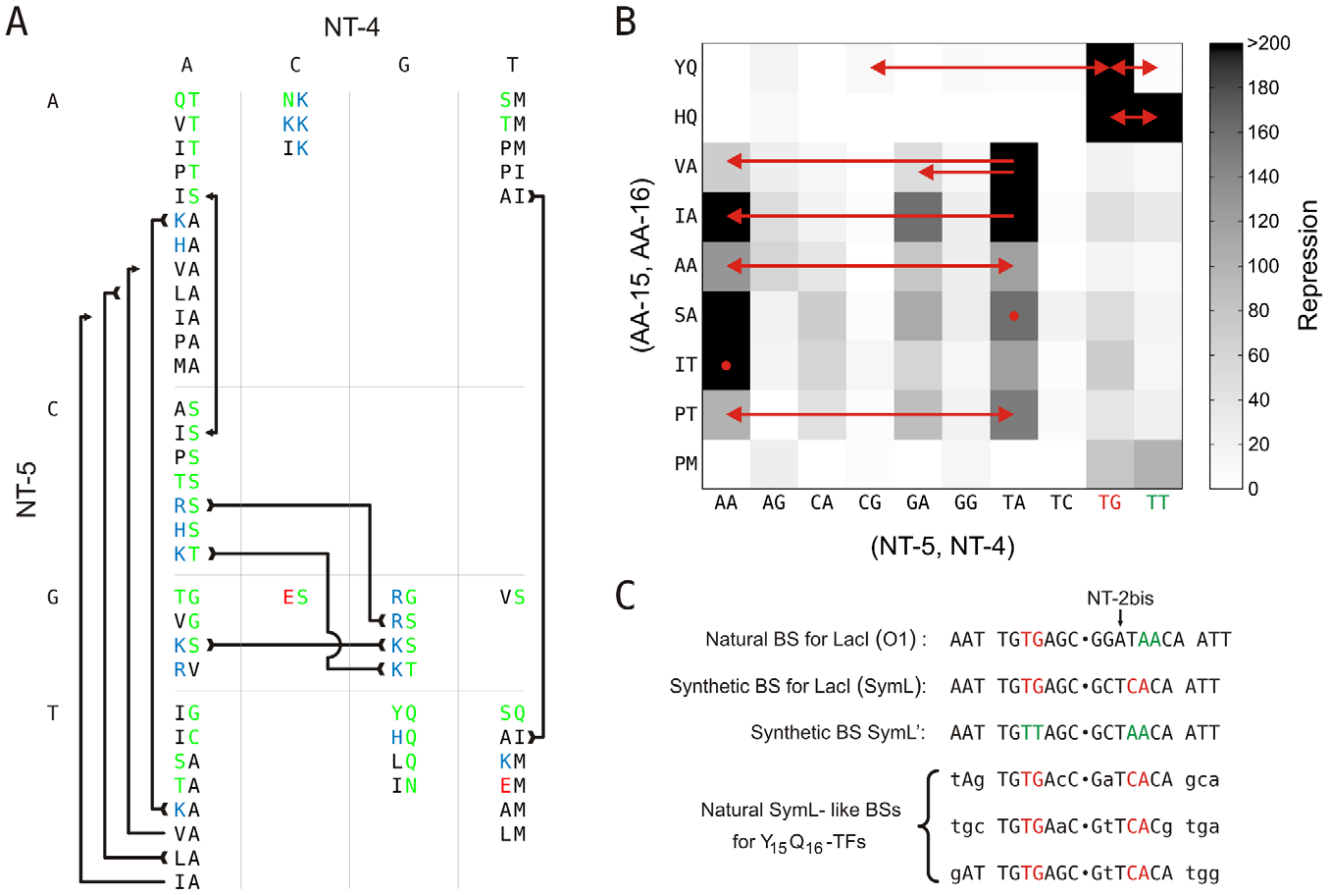
Recognition code and experimental confirmations. A) Sequence correlations between (AA-15, AA-16) and (NT-5, NT-4) extracted from correlations in Table S1. AAs sequences recognizing a same sequence of NTs were grouped. Here, we only considered significant palindromic NT sequences; for example, (NT-5, NT-4) = TG means 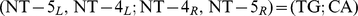. We included the case for (AA-15, AA-16) = YQ corresponding to the synthetic SymL site in C). Recognition degeneracies are represented as unidirectional arrows (asymmetrical intrinsic), bidirectional divergent arrows (symmetrical intrinsic), and bidirectional convergent arrows (extrinsic). Colors for polar (green), basic (blue), acidic (red) and hydrophobic (black) amino acids. B) Agreement between synthetic and natural data. Recognition of (NT-5, NT-4)-palindromes by different (AA-15, AA-16)-LacI mutants (YQ is the wild type). Data from [19] –from which we only considered those sequences (AA-15, AA-16) with a natural correspondence in Table S1. Rest of BS positions as in SymL. The larger the TF/BS affinity, the stronger the repression of the 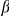-galactosidase activity. Experimental conditions limited repression to a factor of 200. Arrows indicated again degeneracy classes. Predictions for wild type YQ correspond to asymmetric natural BSs (see text). (NT-5, NT-4)-palindromes involved in the predicted correlations for PM (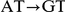, see Table S1) lack an experimental test. Accordingly, PM do not exhibit a strong affinity for any of the tested palindromes (see Fig. S3), C) Natural and synthetic operators. A dot distinguishes the half sites. Flanking nucleotides separated by a space to help visualization of the highly conserved central region of the BSs. Colors identify different palindromic or mixed combinations in the specificity nucleotides (see Table S2 for more details).

We showed a subset of these results, with only significant palindromic combinations, in Fig. 4.A. Recognition sequences were sorted by the left semisequence of the palindromes they recognize, and connected according to their resolved degeneracies. For instance, 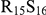 shows an extrinsic degeneracy between (NT-5, NT-4) = CA and (NT-5, NT-4) = GG. The variability of the recognition correlations in AA-15 became manifest also in this figure, a flexibility previously pointed out by mutational studies [19]. Our genomic approach confirmed then that the role of AA-16 as the strongest determinant of specificity applies throughout the TVSR group [19].

Since the general mode of binding in the LacI family involves DNA bending, one could expect that the direct readout of the contacting residues would be strongly conditioned by the characteristics of this specific type of indirect reading [11], [12], [50]. This would directly imply that TFs with the same contacting residues could recognize different NT sequences. However, the small number of extrinsic degeneracies found suggests that the degree of bending remains substantially conserved throughout the TVSR group.

**Figure 5:**
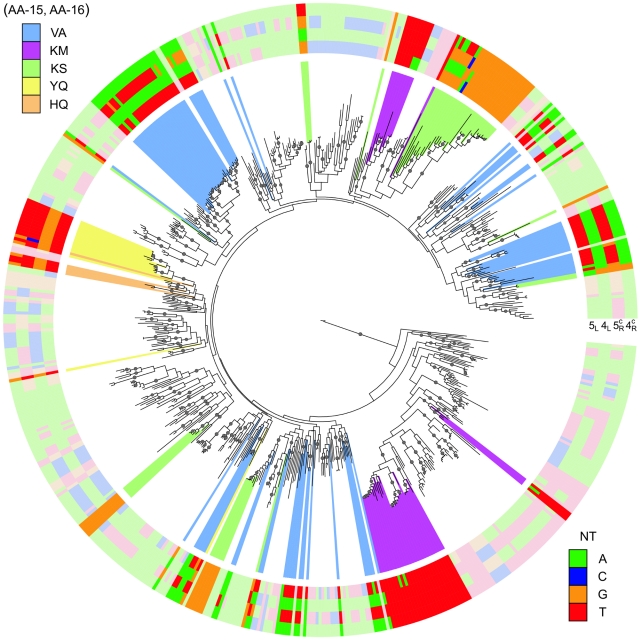
Convergence of binding modes in the gene tree. Gene tree involving all TFs with BSs in Table S1 (623 TFs) plus the 3 TFs with 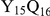 binding to natural SymL-like BSs (Fig. 4.C and Table S2). Only one BS per TF is shown. The external color code displays the specificity-associated positions –to help visualization of palindromic combinations right positions are read in the complementary (*c*) strand: 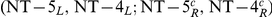. The color background in several branches corresponds to different recognition AAs (only a few recognition classes were enhanced). External color code in these branches shows darker colors to help visualization. Dots in branches denote bootstrap values larger than 80 (for 100 trees total, see Fig. S4 for more details).

The consistent next step after proposing an AA/NT recognition code was to validate its predictions. We approached this issue in the next sections in three complementary ways. First, we compared the theoretical predictions with experimental data from LacI mutants (Fig. 4.B and Fig. S3) [19], [20]. Second, we confirmed the existence of natural counterparts of BSs previously interpreted only as synthetic constructs (Fig. 4.C). Finally, by computing a gene tree including all TFs with at least one BS in Table S1, we identified several convergence events in the recognition process –the same AAs/NTs association in different tree locations (Fig. 5) – that additionally supported the hypothesis of the conservation of the mode of binding, and that overall indicated the presence of a relatively consistent recognition code.

### Mutational studies support code predictions

We compared the theoretical predictions with two experimental studies analysing the DNA binding specificities of *Escherichia coli*'s LacI repressor [19], [20]. Fig. 4.B shows a comparison between the recognition rules in Table S1 and data from the first of these studies, the pioneering work of Müller-Hill and colleagues [19] in which several repressor mutants where isolated and characterized. In this figure, the experimentally measured repression of (NT-5, NT-4)-palindromes by different (AA-15, AA-16)-LacI mutants is shown in boxes (with 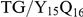 being the wild type interaction), where the theoretical predictions are superimposed. These predictions are indicated by arrows, following Table S1, with dots denoting non-degenerate associations [links to a single (NT-5, NT-4) pair]. The agreement between theory and experiments emphasizes the presence of an intrinsically degenerate code, with the only discrepancy of the wild type 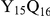.

This inconsistency of the wild type class is due to the difference between the BSs considered in our study and those examined experimentally. Theoretical correlations were derived from natural BSs exhibiting variations over the asymmetric O1 site for *E. coli*'s LacI (Fig. 4.C). This specific BS presents an intervening base (NT-2bis, Fig. 4.C) which introduces an asymmetry between the protein contacts made over the left and right half sites [29], [38]. However, LacI can bind a palindromic BS lacking the intervening nucleotide. This BS is called SymL (Fig. 4.C) because it is synthetically built from the symmetrization of the left half site of O1 [29]. The mutational studies were based on variations over SymL [19] –for example, the SymL' site in Fig. 4.C. In such synthetic constructs the palindromic affinity of LacI is severely restricted to (NT-5, NT-4) = TG. Moreover, LacI is unable to bind the SymL/SymL'-like mixture (Table S2) obtained from the delection of 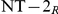 in the natural O1 site [51].

In a more recent work, Lewis and colleagues [20] characterized the associations between a set of 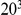 *E. coli*'s LacI mutants for the triplet (AA-15, AA-16, AA-20) –corresponding to the AA coordinates 17, 18 and 22 of LacI, respectively– and the 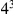 palindromic (NT-6, NT-5, NT-4)-variants of the SymL operator. We plotted in Fig. S3 a comparison between the recognition pairs obtained in these experiments (corresponding to the TVSR group) and the theoretical predictions involving significant NT palindromic combinations (Fig. 4.A). We noticed again a strong agreement between theory and experiment, which becomes more evident when considering that regulators sharing the same AA-16 sequence tend to bind similar NT sequences. Note also that some of the theoretical correlations could remain untested due to the specific mutant sampling of the screening protocol.

Our predictions appeared nevertheless at odds with some experiments done with *lac* family members in the latter study [20]. In this case, the recognition triplet (AA-15, AA-16, AA-20) of LacI was swapped to that of nine different members of the family, i.e., MalR, RbtR, FruR, PurR, RbsR, GalR, CytR, RafR and ScrR (the last four in the TVSR group). The sequence of (NT-6, NT-5, NT-4) in SymL was changed accordingly for these regulators to that of a natural BS in which they were known to bind. Only the mutants associated to GalR and FruR worked [20]. This seemingly contradiction is partly linked to the presence of members out of the TVSR group (see below) and the use of single BSs in the repressor-operator characterization (see Text S1, section 3 for a detailed discussion).

The agreement between the familial (genomic-based) specificity predictions and the corresponding mutational experiments in the TVSR set (Fig. 4.B and Fig. S3), this set being 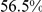 of the whole family, suggests that the preferential binding of arginine in AA-20 to guanine in NT-6 might turn the structural environment under which the recognition partners (AA-15, AA-16)/(NT-5, NT-4) operate with strong stability, so that indirect readouts did not prevent the emergence of a consistent recognition code.

### Code predictions help identify natural correspondences of a synthetic binding mode

The binding of LacI to the synthetic site SymL was believed to be a laboratory construct, not representative of the characteristic binding mode of this regulator [35]. However, two observations from our study supported the presence of a natural counterpart of this synthetic binding mode. First, the natural BSs for the related recognition sequence 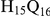 resembled either the perfect palindromic sequences of SymL and SymL', or their mixture (Table S2, see the corresponding logo in Fig. 2.D). Second, although every BS involved in the 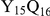 logo in Fig. 2.C incorporated the inserted nucleotide, we also found several BSs related to the synthetic SymL construction (Fig. 4.C and Table S2) in the first BS search based on PF. In agreement with the mutant model [19], [51], neither natural SymL'-like BSs nor mixtures were detected for 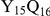 in this PF scan.

That the recognition sequences of 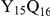
- and 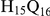-TFs are highly related was also suggested by its location in the gene tree. Fig. 5 shows the gene tree of all TFs with at least one BS in the table of correlations (623 TFs for 811 BSs in Table S1) and the three TFs with 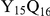 binding to SymL-like BSs. In this tree, branches corresponding to these two recognition classes appeared closely located. In fact, a recent mutational work [52] demonstrated that the 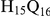 LacI-mutant exhibits a stronger affinity to SymL than the 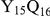 wild type.

### Recognition convergence strengthens structural stability hypothesis

If only a restricted number of specificity determinants (AA to NT pairs) were possible within a particular regulatory family, we should expect instances of convergent evolution for the same recognition AAs in divergent backgrounds. This is indeed what we observed. In the gene tree plotted in Fig. 5 (see also Fig. S4), branches corresponding to several of the largest recognition classes were highlighted. We identified convergence events in the recognition process (i.e., same AAs associated to the same NTs throughout the tree). These findings validated the initial hypothesis that the binding mode was highly conserved and that, as a consequence, evolution finds the same solutions repeatedly (the presence of relatively consistent recognition rules). Such structural stability of the TVSR set could apply to other regulator families.

### Conclusions

This work reveals the first comprehensive resolution of a recognition code for a large group of proteins within a family of transcriptional regulators. This resolution is based on the use of comparative genomics [15], the identification of local transcriptional regulation as a fundamental regulatory architecture in prokaryotes [30]–[34] and the hypothesis of the stability –in the large phylogenetic distances considered– of the domain structure around the recognition sites [10], [14].

This last hypothesis is confirmed by the patterns of differential residue and BS conservation obtained. Indeed, we only found a few instances of TFs that would invalidate our conjecture, i.e., TFs with the same sequence in the specificity pair (AA-15, AA-16) but recognizing incompatible BSs (extrinsic degeneracies). Moreover, the convergence events and the agreement of the correlations with mutational data (including the extension of the rule of the AA-15 flexibility to become a dominant family attribute) support the assumption that the mode of binding is conserved for a large fraction of the family.

A few caveats to our approach should be noticed. First, we considered a stringent protocol to select for BSs. This method combined PF, iterated PWM refinement, and further removal of BSs with potential spurious nucleotides exhibiting no special affinity (see Text S1, section 2). In this way, those AA/NT relationships incorporated into the code should exhibit at least a minimal moderate affinity. Of course, any false positive removal is made at the cost of losing some true positives. An example of this was the loss of the BS for RafR [26], which was detected in the initial PF search but removed after the processing protocol. In any case, this was a consequence of the dominance within the TVSR set of a canonical mode of binding associated to an ideal BS backbone given by the conserved pattern (T)G–A-CG-T–C(A) in Fig. 2.E. A second limitation to our approach is the reliability of the extrinsic/intrinsic degeneracy analysis. The most reliable ones correspond to TF classes with many members and many detected BSs, e.g., the TF class corresponding to 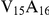 (see the Appendix in Text S1). This second limitation could be overcome as more genomes become available.

In contrast to what appears to happen with the LacI family as a whole [20], the natural recognition correlations within the TVSR subfamily could be largely reproduced by mutational experiments. Thus, the genomically-derived correlations will be useful to complete the specificity map derived with mutational approaches only [19], [20]. Moreover, the use of natural correlations will be probably essential to guide the redesign of a library of regulators that can target the maximal number of arbitrary sequences in the non-conserved positions of the consensus sequence. Note that, beyond the code established between the pairs (AA-15, AA-16) and (NT-5, NT-4), the mutual information analysis of Fig. S1 suggested that there existed alternative AA and NT positions also involved in specificity tasks. In particular, the sequence in NT-2 was associated in this analysis to those of AA-5, AA-15 and AA-55. The same applies for a mutual information analysis restricted to the TVSR set (data not shown). This specificity role of AA-55 was demonstrated in the particular case of the purine repressor [41]. As AA-15 could be coupling the recognition of NT-2 to that of the pair (NT-5, NT-4), the resolution of the specificity map for the triad (NT-5, NT-4, NT-2) could be beyond the scope of any mutational approach without a previous genomic blueprint.

In summary, the main advantage of the BS search based on local regulation is its potential applicability to any annotated genome and TF family, without the limitations linked to orthology and functionality definitions, i.e., the functional relationship between the TF and the regulated operon trivially exists in the case of autoregulation. The explicit correlations obtained in this analysis can thus be refined with sequence data from newly sequenced genomes, and could ultimately act as a blueprint for the synthetic redesign of TFs with new specificities. These correlations constitute the first candidate to a relatively consistent recognition code applicable to an extensive subfamily of transcriptional regulators.

## Methods

### Selection of sequences for HTH-LacI domains

5597 AA sequences for HTH-LacI domains (Smart SM00354) were obtained from MicrobesOnline [36]. The median length value of this domain (including both the HTH and hinge-helix regions) is 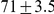 AAs. To guarantee the functionality of the domains, we selected from the starting set every sequence whose length is inside the range of 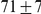 AAs, and removed those lacking the 26-AA Pfam domain PF00356 –this label corresponds to the HTH core of the HTH-LacI domain. We also discarded three cases of proteins containing two SM00354 domains. Finally, we removed overrepresented sequences due to strain variations in the database to get a final set of 2639 sequences.

### Domain alignment

We use Muscle [53] to add each of the HTH-LacI domains to a previous Smart curated alignment involving 49 SM00354 domains [54]. After the removal of columns exhibiting gaps in more than 80% of its sequences, we obtained a seed-alignment with 71 AA positions. Then, for each of the 2639 sequences we applied the following protocol: i) the sequence is added to the seed-alignment using the mentioned option of Muscle; ii) all those positions that imply the insertion of a gap in the seed-alignment are removed from the sequence; and iii) the sequence (in its aligned configuration) is removed from the seed-alignment and saved. After the process was completed, none of the 71 positions in the final alignment of the 2639 domains (Fig. 1.C) exhibited gaps in more than 5% of sequences. We extracted all the recognition helix sequences from the alignment. 1490 out of 2639 domains belonged to the TVSR group (Dataset S1).

### Selection of intergenic regions for BS search

We could extract from the operons predictions included in MicrobesOnline [36] the non-coding region located upstream of the operon encoding the HTH-LacI domain (up to 200 bp), and also the non-coding region located before the downstream neighbor operon (Fig. 2.A, Dataset S1). When the regulated operon is located downstream of the regulator, both operons are usually encoded in the same strand (unidirectional architecture [31]). Thus, in the case of downstream regulation we only considered the unidirectional orientation –this occurs in 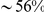 of domains. We did not included alternative convergent orientation (downstream operon encoded in the opposite strand) because under this architecture neighbor regulation is much less common [31]. Sequences were truncated if the next upstream coding region was reached (Fig. 2.A, red lines). From every region we also obtained an extended version of 250 bp that includes the range of coding positions from +1 to +50. These extended regions were never truncated (Fig. 2.A, green lines).

### Recognition TF classes and first BS search by PF

Within the TVSR group we divided the intergenic regions in groups associated to domains sharing the same (AA-15, AA-16) sequence. On each group (recognition class), we made a first BS scan using PF techniques as implemented in the Gibbs Motif Sampler [55], with the following parameters: estimated total number of BSs in a given group of regions equals the number of these regions; one BS per region at the most; palindromic BSs of 14 bp without fragmentation. Results were robust to changes in these parameters, including the estimated BS length and the palindromic nature of the sites. To avoid circularity we did use uninformed priors based on the average background composition [56]. The PF scan was applied over the truncated version of the intergenic regions to avoid coding zones, which, as it happens with BSs, are more conserved than the non-functional intergenic sequences. Finally, we discarded BSs with confidences below 40%.

### Second BS search by PWM

After the first BS scan we had at most one BS per intergenic region. We refined and extended our results through an iterative process of PWM construction and BS selection. This time, we considered that there might be multiple BSs per intergenic region and BSs located in the coding zone. Firstly, we built a PWM from the BSs found in the PF scan using a constant pseudocount function 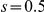 [49] (results were robust under variations on this parameter). Secondly, we slided this PWM over the extended version of the intergenic regions and generously selected all those sites with a score over the minimal one in the starting BS set. The sites selected in this search is what we called the candidate sites. Finally, we applied the following protocol to look for the most significant candidates: i) generation of a null set of 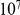 scores that was obtained by sliding the PWM over random versions of the intergenic regions; ii) selection of every candidate whose score had a p-value below 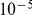 when compared to the null set; iii) construction of a new PWM from the candidates selected in ii); and iv) computation of the score for all candidates under the new PWM.

Using the new PWM to generate a new null set, these four steps were iterated until convergence. The resulting set of 942 BSs was the end product of the whole process of search (Dataset S1). All the found BSs exhibited Z-scores above 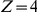. Each BS was read in the sense strand –consequently, its left and right semisequences were univocally determined. See Text S1, section 1 and Fig. S5 for a comparison with more standard approaches to BS search.

### Consensus logo

We extracted the consensus sequence of BSs associated to a same recognition class and then aligned the whole set of consensus sequences to obtain the consensus logo (Fig. 2.E). Using the alignment of consensus sequences instead of the raw alignment of all found BSs avoids the over-representation of those BSs corresponding to the most populated classes. The raw alignment exhibited the same qualitative behavior to that of Fig. 2.E.

### Identification and classification of degeneracies

We successively applied the following protocol to each set of BSs associated with the same recognition AAs (see section 2 of Text S1, Table S3, Fig. S6, and Fig. S7 for a more detailed description). First, a triangular matrix *F* containing the frequencies of the 136 possible combinations for the quartet of positions 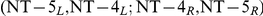 was computed. Second, a matrix *S* was extracted from *F* by selecting combinations found to be statistically significant (with respect to those observed in the genomic background). Third, significantly under-represented mixtures were identified in *S*, as the absence of mixed combinations is linked to extrinsic degeneracies (Fig. 3.B, right). Finally, each extrinsic degeneracy partitioned *S* into two submatrices in which the two types of intrinsic degeneracy were resolved. In the absence of any significant high frequency in a submatrix we kept the symmetrical recognition scenario of the null model (Fig. 3.B, left). Moreover, the presence of a significant frequency usually corresponded to a palindromic combination. In this case, we considered an asymmetrical recognition process with a dominant palindrome (Fig. 3.B, center).

### Gene tree

The full AA-sequences of the 626 TFs with at least a BSs in Table S1 (623 TFs) plus the 3 TFs with 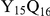 binding to natural SymL-like BSs (Fig. 4.C and Table S2) were aligned and refined with Muscle. This alignment was trimmed with Gblocks [57]. Finally, we use PhyML to build the tree in Fig. 5. Supplementary Fig. S4 contains a more detailed version of this tree in which each protein is labeled with its VIMSS ID plus the recognition AA pair. In this larger version, we plotted all the BSs associated to each TF (we found four BSs per TF at most).

## Supporting Information

Dataset S1 Sequences of proteins, domains, intergenic regions and binding sites.
(0.67 MB ZIP)

Figure S1 Mutual information (covariance dependency) values between 370 domains in our alignment for which we could univocally associate BSs in RegTransBase v5 (reference [46], main text) and the alignment of these BSs (see Text S1, section 1 for details on the use of RegTransBase data). Logos for these alignments are explicitely shown. The global mutual information pattern reflects the symmetrical nature of the contacts made by the monomers over the corresponding half site. Mutual information analyses cannot solve interactions between highly conserved NT and/or AA positions -note how they correspond to the darkest rows and columns (see reference [47], main text). This is the case of the links between the hinge-helix AA-51 and AA-54 with the central CG group. On the other hand, the largest covariances for NT-5 corresponded to AA-15 and AA-16. Although several AAs (like AA-15) exhibited appreciable scores for NT-4, the maximal mutual information is obtained with AA-16. NT-6 is strongly correlated with AA-20, with no more appreciable correlations for these NT and AA coordinates. Finally, NT-2 is correlated, in decreasing order of importance, with AA-55, AA-15 and AA-5.
(0.50 MB TIF)

Figure S2 Conserved positions in recognition helix.
(0.02 MB PDF)

Figure S3 Comparison of theoretical predictions with experimental data. Black boxes correspond to (AA-15, AA-16)/(NT-5, NT-4) binding partners in a protocol of phenotype screening of binding mutants (reference [20], main text). Vertical gray lines separate groups of amino acid sequences sharing the same AA-16. Green dots indicate the theoretical sequence correlations [involving significant (NT-5, NT-4)-palindromes, see Fig. 4.A in main text]. One should consider in this comparison that: i) regulators sharing the same AA-16 sequence tend to bind similar nucleotide sequences, and ii) due to the sampling effects of the screening method, some of the theoretical recognition predictions remained possibly untested. The main discrepancy observed corresponded to those regulators with a methionine in AA-16, where we found a consistent signal of binding to (NT-5, NT-4) = TT which is abstent in the mutational experiment. This trend was however in agreement with the experimental data reported in reference [19], main text. Note also here the considerable number of mutants that were still able to bind the wild type sequence of SymL, (NT-5, NT-4) = TG.
(0.16 MB TIF)

Figure S4 Full version of the gene tree in Figure 5, main text. This tree involves the same transcriptional factors (TFs) of the simplified tree; however, we plotted now all the binding sites (BSs) associated to each TF (we found four BSs per TF at most). Each external quartet of colored boxes corresponds to the specificity-associated positions of one BS -to help visualization of palindromic combinations, right positions are read in the complementary (*c*) strand: (NT-5_L_, NT-4_L_; NT-5*^c^*_R_, NT-4*^c^*_R_). The color background in several branches corresponds to different recognition amino acids (only a few recognition classes were enhanced). Dots in branches denote bootstrap values larger than 80 (for 100 trees total).
(3.33 MB TIF)

Figure S5 Comparison with RegTransBase.
(0.03 MB PDF)

Figure S6 Example of matrix *F*.
(0.04 MB PDF)

Figure S7 Protocol to distinguish among the different types of degeneracies.
(0.02 MB PDF)

Table S1 Table of correlations.
(0.03 MB PDF)

Table S2 Natural and synthetic operators for Y_15_Q_16_ and H_15_Q_16_.
(0.03 MB PDF)

Table S3 Examples of two-strand-detailed binding sites.
(0.02 MB PDF)

Text S1 Efficiency of the BS search method based on local regulation. Resolution of the recognition correlations. Comparison with mutational data. Glossary. Appendix: BS logos.
(1.45 MB PDF)

## References

[1] Seeman NC, Rosenberg JM, Rich A (1976). Sequence-specific recognition of double helical nucleic acids by proteins.. Proc Natl Acad Sci U S A.

[2] Pabo CO, Sauer RT (1984). Protein-DNA recognition.. Annu Rev Biochem.

[3] Desjarlais JR, Berg JM (1992). Toward rules relating zinc finger protein sequences and DNA binding site preferences.. Proc Natl Acad Sci U S A.

[4] Suzuki M, Brenner SE, Gerstein M, Yagi N (1995). DNA recognition code of transcription factors.. Protein Eng Des Sel.

[5] Choo Y, Klug A (1997). Physical basis of a protein-DNA recognition code.. Current Opinion In Struct Biol.

[6] Matthews BW (1988). Protein-DNA interaction. No code for recognition.. Nature.

[7] Pabo CO, Nekludova L (2000). Geometric analysis and comparison of protein-DNA interfaces: Why is there no simple code for recognition?. J Mol Biol.

[8] Benos PV, Lapedes AS, Stormo GD (2002). Probabilistic code for DNA recognition by proteins of the EGR family.. J Mol Biol.

[9] Sarai A, Kono H (2005). Protein-DNA recognition patterns and predictions.. Annu Rev Biophys Biomol Struct.

[10] Maerlk SJ, Quake SR (2009). Experimental determination of the evolvability of a transcription factor.. Proc Natl Acad Sci U S A.

[11] Gromiha MM, Siebers JG, Selvaraj S, Kono H, Sarai A (2004). Intermolecular and intramolecular readout mechanisms in protein-DNA recognition.. J Mol Biol.

[12] Paillard G, Lavery R (2004). Analyzing protein-DNA recognition mechanisms.. Struct.

[13] Espinosa Angarica V, Gonzalez Perez A, Vasconcelos AT, Collado-Vides J, Contreras-Moreira B (2008). Prediction of TF target sites based on atomistic models of protein-DNA complexes.. BMC Bioinformatics.

[14] Hall BM, LeFevre KR, Cordes MHJ (2005). Sequence correlations between Cro recognition helices and cognate O-R consensus half-sites suggest conserved rules of protein-DNA recognition.. J Mol Biol.

[15] Desai TA, Rodionov DA, Gelfand MS, Alm EJ, Rao CV (2009). Engineering transcription factors with novel DNA-binding specificity using comparative genomics.. Nucleic Acids Res.

[16] Luscombe NM, Laskowski RA, Thornton JM (2001). Amino acid-base interactions: a three-dimensional analysis of protein-DNA interactions at an atomic level.. Nucleic Acids Res.

[17] Wolfe SA, Grant RA, Elrod-Erickson M, Pabo CO (2001). Beyond the “recognition code”: structures of two Cys2His2 zinc finger/TATA box complexes.. Struct.

[18] Morozov AV, Havranek JJ, Baker D, Siggia ED (2005). Protein-DNA binding specificity predictions with structural models.. Nucleic Acids Res.

[19] Sartorius J, Lehming N, Kisters B, von Wilcken-Bergmann B, Müller-Hill B (1989). *lac* repressor mutants with double or triple exchanges in the recognition helix bind specifically to lac operator variants with multiple exchanges.. EMBO J.

[20] Milk L, Daber R, Lewis M (2010). Functional rules for lac repressor-operator associations and implications for protein-DNA interactions.. Protein Science.

[21] Smith GP (1991). Surface presentation of protein epitopes using bacteriophage expression systems.. Curr Opin Biotechnol.

[22] Nardelli J, Gibson T, Charnay P (1992). Zinc finger-DNA recognition: analysis of base specificity by site-directed mutagenesis.. Nucleic Acids Res.

[23] Sandelin A, Wasserman WW (2004). Constrained binding site diversity within families of transcription factors enhances pattern discovery bioinformatics.. J Mol Biol.

[24] Mahony S, Auron PE, Benos PV (2007). DNA familial binding profiles made easy: comparison of various motif alignment and clustering strategies.. PLoS Comput Biol.

[25] Sera T (2009). Zinc-finger-based artificial transcription factors and their applications.. Adv Drug Deliv Rev.

[26] Weickert MJ, Adhya S (1992). A family of bacterial regulators homologous to Gal and Lac repressors.. J Biol Chem.

[27] Moscou MJ, Bogdanove AJ (2009). A simple cipher governs DNA recognition by TAL effectors.. Science.

[28] Boch J, Scholze H, Schornack S, Landgraf A, Hahn S (2009). Breaking the code of DNA binding specificity of TAL-type III effectors.. Science.

[29] Lewis M (2005). The *lac* repressor.. C R Biol.

[30] Korbel JO, Jensen LJ, von Mering C, Bork P (2004). Analysis of genomic context: prediction of functional associations from conserved bidirectionally transcribed gene pairs.. Nat Biotechnol.

[31] Warren PB, ten Wolde PR (2004). Statistical analysis of the spatial distribution of operons in the transcriptional regulation network of *Escherichia coli*.. J Mol Biol.

[32] Hershberg R, Yeger-Lotem E, Margalit H (2005). Chromosomal organization is shaped by the transcription regulatory network.. Trends Genet.

[33] Kolesov G, Wunderlich Z, Laikova ON, Gelfand MS, Mirny LA (2007). How gene order is influenced by the biophysics of transcription regulation.. Proc Natl Acad Sci U S A.

[34] Camas FM, Poyatos JF (2008). What determines the assembly of transcriptional network motifs in *Escherichia coli*?. PLoS ONE.

[35] Perros M, Steitz T (1996). DNA looping and Lac repressor-CAP interaction [comment on “Crystal structure of the lactose operon repressor and its complexes with DNA and inducer”].. Science.

[36] Alm EJ, Huang KH, Price MN, Koche RP, Keller K (2005). The MicrobesOnline web site for comparative genomics.. Genome Res.

[37] Lewis M, Chang G, Horton NC, Kercher MA, Pace HC (1996). Crystal structure of the lactose operon repressor and its complexes with DNA and inducer.. Science.

[38] Kalodimos CG, Boelens R, Kaptein R (2004). Toward an integrated model of protein-DNA recognition as inferred from NMR studies on the Lac repressor system.. Chem Rev.

[39] Salinas RK, Folkers GE, Bonvin AMJJ, Das D, Boelen R (2005). Altered specificity in DNA binding by the lac repressor: A mutant lac headpiece that mimics the gal repressor.. ChemBioChem.

[40] Schumacher MA, Choi KY, Zalkin H, Brennan RG (1994). Crystal-structure of LacI member, PurR, bound to DNA: minor-groove binding by *α*-helices.. Science.

[41] Glasfeld A, Koehler AN, Schumacher MA, Brennan RG (1999). The role of lysine 55 in determining the specificity of the purine repressor for its operators through minor groove interactions.. J Mol Biol.

[42] Schumacher MA, Allen GS, Diel M, Seidel G, Hillen W (2004). Structural basis for allosteric control of the transcription regulator CcpA by the phosphoprotein HPr-Ser46-P.. Cell.

[43] Bell CE, Lewis M (2001). The Lac repressor: a second generation of structural and functional studies.. Curr Opin Struct Biol.

[44] Jørgensen CI, Kallipolitis BH, Valentin-Hansen P (1998). DNA-binding characteristics of the *Escherichia coli* CytR regulator: a relaxed spacing requirement between operator half-sites is provided by a flexible, unstructured interdomain linker.. Mol Microbiol.

[45] Francke C, Kerkhoven R, Wels M, Siezen RJ (2008). A generic approach to identify transcription factor-specific operator motifs; inferences for LacI-family mediated regulation in *Lactobacillus plantarum* WCFS1.. BMC Genomics.

[46] Kazakov AE, Cipriano MJ, Novichkov PS, Minovitsky S, Vinogradov DV (2007). RegTransBase - a database of regulatory sequences and interactions in a wide range of prokaryotic genomes.. Nucleic Acids Res.

[47] Mahony S, Auron PE, Benos PV (2007). Inferring protein–DNA dependencies using motif alignments and mutual information.. Bioinformatics.

[48] Ureta-Vidal A, Ettwiller L, Birney E (2003). Comparative genomics: Genome-wide analysis in metazoan eukaryotes.. Nat Rev Genet.

[49] Wasserman WW, Sandelin A (2004). Applied bioinformatics for the identification of regulatory elements.. Nat Rev Genet.

[50] Baldi P, Baisnée P (2000). Sequence analysis by additive scales: DNA structure for sequences and repeats of all lenghts.. Bioinformatics.

[51] Betz JL, Sasmor HM, Buck F, Insley MY, Caruthers MH (1986). Base substitution mutants of the *lac* operator - invivo and invitro affinities for *lac* repressor.. Gene.

[52] Lewis M, Daber R (2009). Towards evolving a better repressor.. Protein Eng Des Sel.

[53] Edgar RC (2004). MUSCLE: multiple sequence alignment with high accuracy and high throughput.. Nucleic Acids Res.

[54] Schultz J, Copley RR, Doerks T, Ponting CP, Bork P (2000). SMART: a web-based tool for the study of genetically mobile domains.. Nucleic Acids Res.

[55] Thompson W, Rouchka EC, Lawrence CE (2003). Gibbs Recursive Sampler: finding transcription factor binding sites.. Nucleic Acids Res.

[56] Bergman NH (2007). Methods in Molecular Biology, vol. 395: Comparative Genomics, Volume 1, Chapter 25.

[57] Castresana J (2000). Selection of conserved blocks from multiple alignments for their use in phylogenetic analysis.. Mol Biol Evol.

