# Supplementary figures and images for: Local Gene Regulation Details a Recognition Code within the LacI Transcriptional Factor Family
Source: PLoS Comput Biol. 2010 Nov 11;6(11):e1000989. doi: 10.1371/journal.pcbi.1000989 (PMC2978694; doi:10.1371/journal.pcbi.1000989)

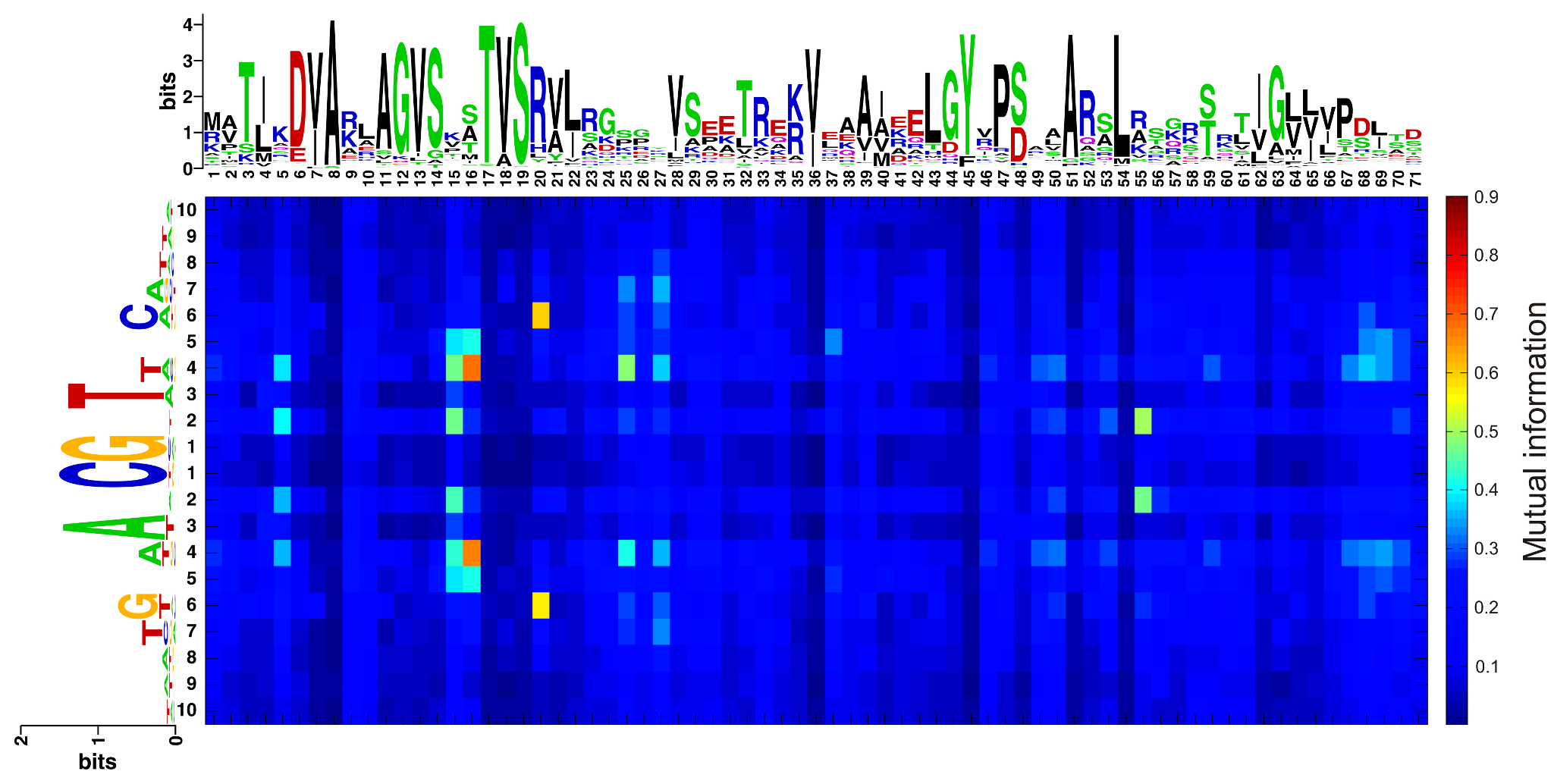

Supplement: Figure S1 — Mutual information (covariance dependency) values between 370 domains in our alignment for which we could univocally associate BSs in RegTransBase v5 (reference [46], main text) and the alignment of these BSs (see Text S1, section 1 for details on the use of RegTransBase data). Logos for these alignments are explicitely shown. The global mutual information pattern reflects the symmetrical nature of the contacts made by the monomers over the corresponding half site. Mutual information analyses cannot solve interactions between highly conserved NT and/or AA positions -note how they correspond to the darkest rows and columns (see reference [47], main text). This is the case of the links between the hinge-helix AA-51 and AA-54 with the central CG group. On the other hand, the largest covariances for NT-5 corresponded to AA-15 and AA-16. Although several AAs (like AA-15) exhibited appreciable scores for NT-4, the maximal mutual information is obtained with AA-16. NT-6 is strongly correlated with AA-20, with no more appreciable correlations for these NT and AA coordinates. Finally, NT-2 is correlated, in decreasing order of importance, with AA-55, AA-15 and AA-5. (0.50 MB TIF) [file pcbi.1000989.s002.tif]

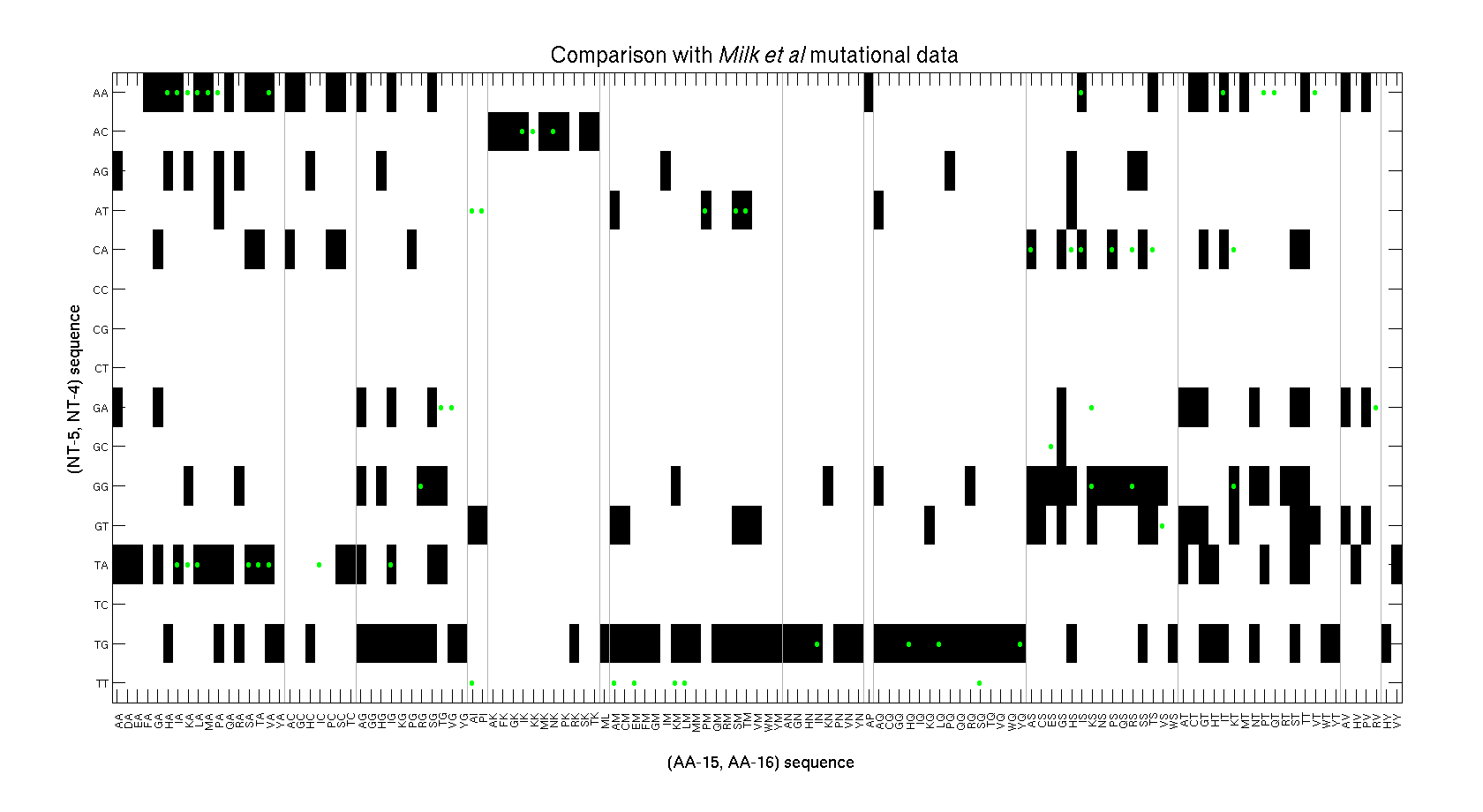

Supplement: Figure S3 — Comparison of theoretical predictions with experimental data. Black boxes correspond to (AA-15, AA-16)/(NT-5, NT-4) binding partners in a protocol of phenotype screening of binding mutants (reference [20], main text). Vertical gray lines separate groups of amino acid sequences sharing the same AA-16. Green dots indicate the theoretical sequence correlations [involving significant (NT-5, NT-4)-palindromes, see Fig. 4.A in main text]. One should consider in this comparison that: i) regulators sharing the same AA-16 sequence tend to bind similar nucleotide sequences, and ii) due to the sampling effects of the screening method, some of the theoretical recognition predictions remained possibly untested. The main discrepancy observed corresponded to those regulators with a methionine in AA-16, where we found a consistent signal of binding to (NT-5, NT-4) = TT which is abstent in the mutational experiment. This trend was however in agreement with the experimental data reported in reference [19], main text. Note also here the considerable number of mutants that were still able to bind the wild type sequence of SymL, (NT-5, NT-4) = TG. (0.16 MB TIF) [file pcbi.1000989.s004.tif]

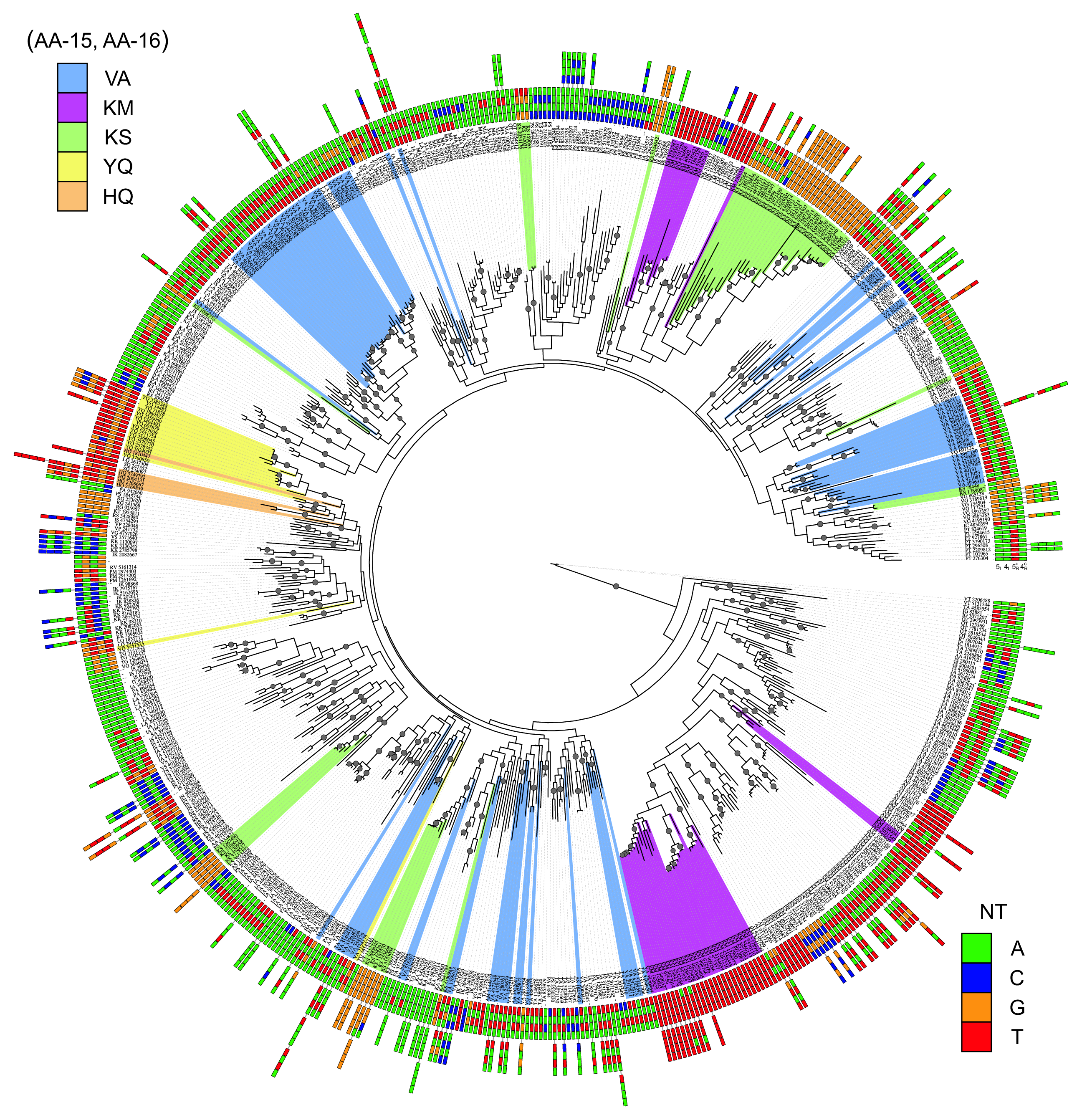

Supplement: Figure S4 — Full version of the gene tree in Figure 5, main text. This tree involves the same transcriptional factors (TFs) of the simplified tree; however, we plotted now all the binding sites (BSs) associated to each TF (we found four BSs per TF at most). Each external quartet of colored boxes corresponds to the specificity-associated positions of one BS -to help visualization of palindromic combinations, right positions are read in the complementary (c) strand: (NT-5L, NT-4L; NT-5c R, NT-4c R). The color background in several branches corresponds to different recognition amino acids (only a few recognition classes were enhanced). Dots in branches denote bootstrap values larger than 80 (for 100 trees total). (3.33 MB TIF) [file pcbi.1000989.s005.tif]
