# Supplementary material for: Local Gene Regulation Details a Recognition Code within the LacI Transcriptional Factor Family
Source: PLoS Comput Biol. 2010 Nov 11;6(11):e1000989. doi: 10.1371/journal.pcbi.1000989 (PMC2978694; doi:10.1371/journal.pcbi.1000989)
Supplement: Figure S2 — Conserved positions in recognition helix. (0.02 MB PDF) [file pcbi.1000989.s003.pdf]

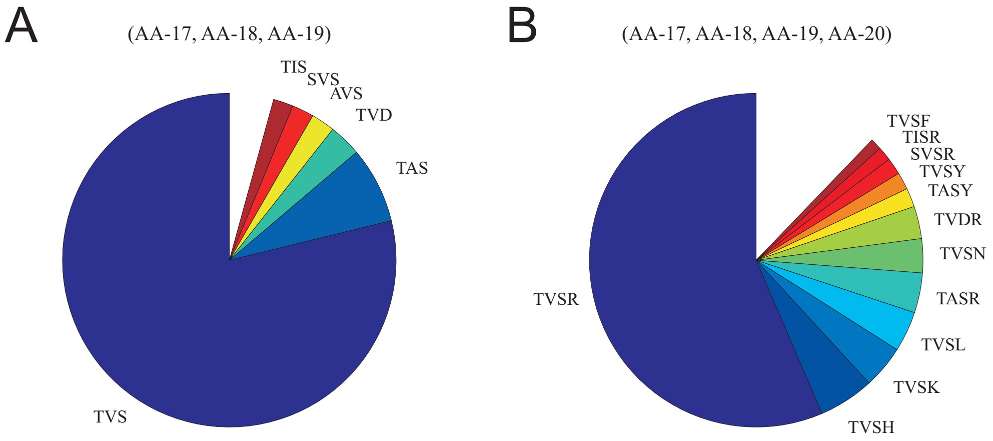

Figure S2: A) Sequence distribution for the triad of recognition helix positions (AA-17, AA-18, AA-19). Only sequences present in more than 1% of domains are showed. B) Idem for the quartet (AA-17, AA-18, AA-19, AA-20).
