# Supplementary material for: Local Gene Regulation Details a Recognition Code within the LacI Transcriptional Factor Family
Source: PLoS Comput Biol. 2010 Nov 11;6(11):e1000989. doi: 10.1371/journal.pcbi.1000989 (PMC2978694; doi:10.1371/journal.pcbi.1000989)
Supplement: Figure S5 — Comparison with RegTransBase. (0.03 MB PDF) [file pcbi.1000989.s006.pdf]

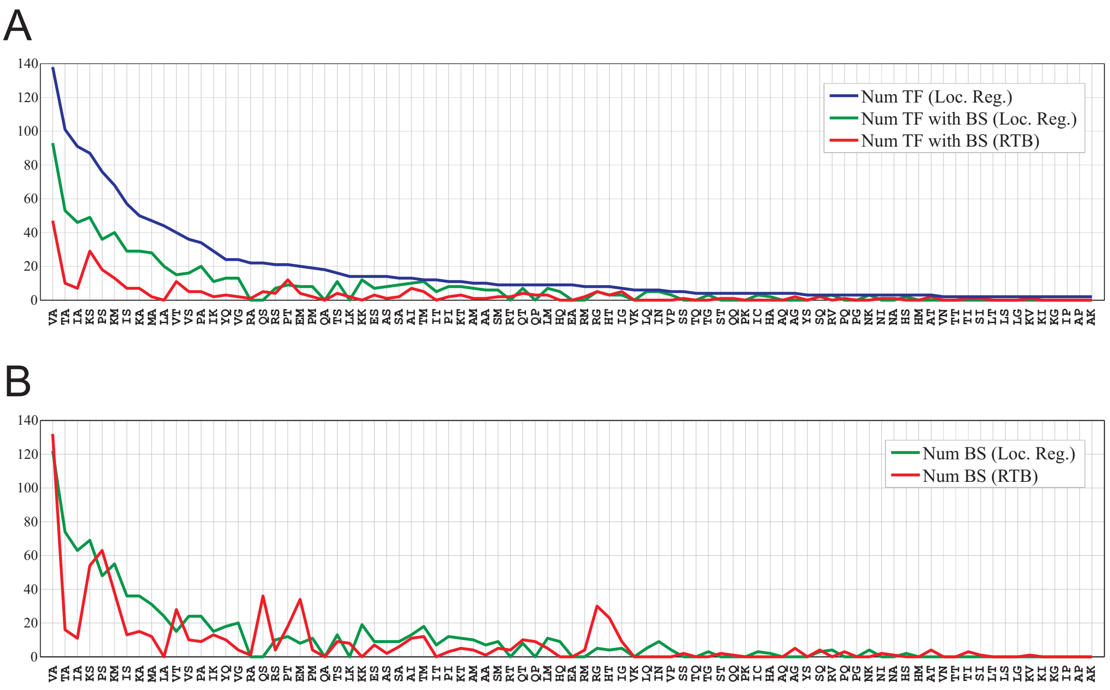

Figure S5: Comparison with RegTransBase (RTB) version v5. Here we only consider domains with the TVSR sequence. Data are detailed by domains with a same sequence in (AA-15, AA-16). Lines to help visualization. A) Blue line: number of TFs in our data set (Loc. Reg., local regulation); green line: number of these for which at least a BS was found; red line: number of TFs in RTB –in this database TFs are always associated to BSs. B) Total number of BSs (green line: this work, red line: RTB).
