# Supplementary material for: Local Gene Regulation Details a Recognition Code within the LacI Transcriptional Factor Family
Source: PLoS Comput Biol. 2010 Nov 11;6(11):e1000989. doi: 10.1371/journal.pcbi.1000989 (PMC2978694; doi:10.1371/journal.pcbi.1000989)
Supplement: Figure S6 — Example of matrix F. (0.04 MB PDF) [file pcbi.1000989.s007.pdf]

semisequence 2

semisequence 1

	AA	AC	AG	AT	CA	CC	CG	CT	GA	GC	GG	GT	TA	TC	TG	TT
AA	0	0	0	0	0	0	0	0	0	0	0	0	0	0	0	0
AC		0	0	0	0	0	0	0	0	0	0	0	0	0	0	0
AG			0	0	0	0	0	0	0	0	4	0	0	0	0	0
AT				0	0	0	0	0	0	0	0	0	0	0	0	0
CA					0	0	0	0	0	0	4	0	0	0	0	0
CC						0	0	0	0	0	0	0	0	0	0	0
CG							0	0	0	0	1	0	0	0	0	0
CT								0	0	0	0	0	0	0	0	0
GA									4	0	2	8	0	0	0	0
GC										0	0	0	0	0	0	0
GG											44	2	0	0	0	0
GT												0	0	0	0	0
TA													0	0	0	0
TC														0	0	0
TG															0	0
TT																0

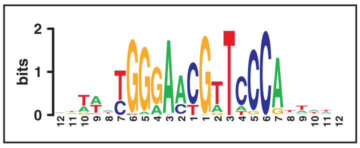

Figure S6: Example of matrix F containing the frequencies of the 136 possible states for positions NT-4 and NT-5 in both semisequences. Due to the equivalence between complementary combinations (see Text S1), semisequences 1 and 2 do not necessarily correspond to the left and right ones, respectively. This matrix is that associated to the recognition amino acids $K_{15}S_{16}$ (BS-logo in insert, all logos in Appendix of Text S1). In red those statistically significant frequencies. Columns and rows corresponding to the semisequences involved in at least one significant state are highlighted in soft gray. Crossing cells (in dark gray) constitute the triangular matrix S (see Text S1 for details).
