# Supplementary material for: Local Gene Regulation Details a Recognition Code within the LacI Transcriptional Factor Family
Source: PLoS Comput Biol. 2010 Nov 11;6(11):e1000989. doi: 10.1371/journal.pcbi.1000989 (PMC2978694; doi:10.1371/journal.pcbi.1000989)
Supplement: Figure S7 — Protocol to distinguish among the different types of degeneracies. (0.02 MB PDF) [file pcbi.1000989.s008.pdf]

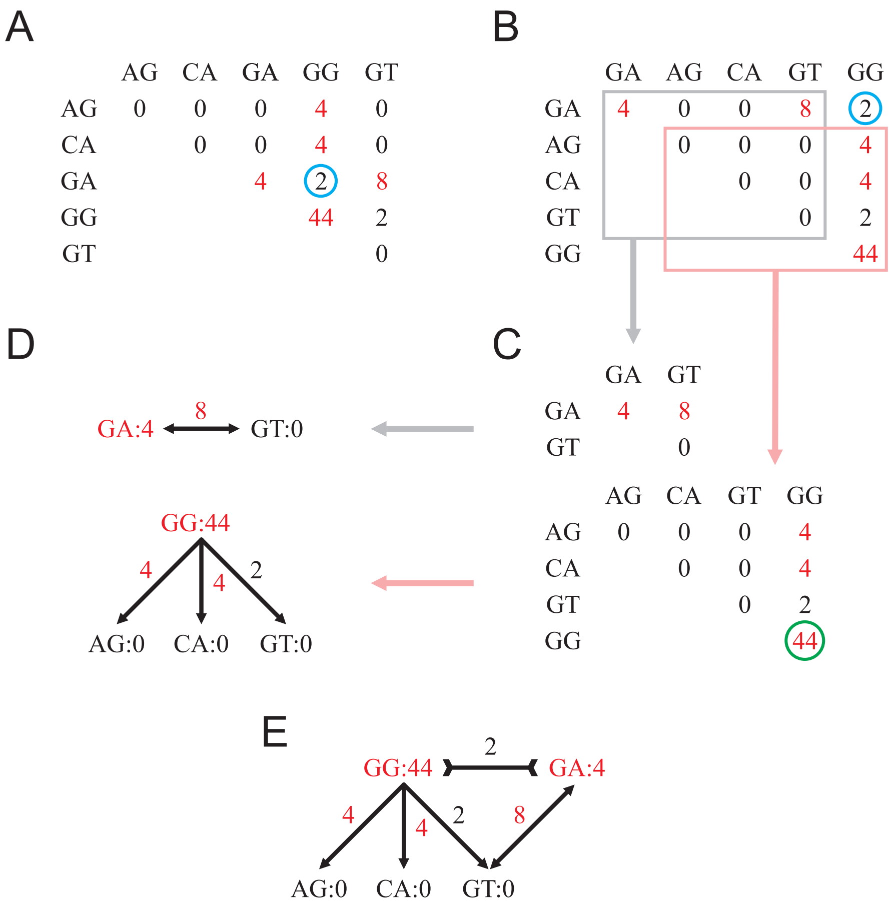

Figure S7: Protocol to distinguish among the different types of degenerate recognitions in the set of binding sites associated to a same recognition sequence ($K_{15}S_{16}$ in this example). See Text S1 for details.
