# Supplementary material for: Local Gene Regulation Details a Recognition Code within the LacI Transcriptional Factor Family
Source: PLoS Comput Biol. 2010 Nov 11;6(11):e1000989. doi: 10.1371/journal.pcbi.1000989 (PMC2978694; doi:10.1371/journal.pcbi.1000989)
Supplement: Table S1 — Table of correlations. (0.03 MB PDF) [file pcbi.1000989.s009.pdf]

Table S1: Correlations between the pair of recognition amino acids (AA-15, AA-16) and their cognate nucleotides (NT-5, NT-4). Each cell in the table corresponds to a particular recognition class. Graphs illustrate degeneracy patterns. Specifically, each node in the graphs corresponds to a palindromic sequence, from which we only annotated the left semisequence. For example, a node denoted AC:10 describes the palindromic combination (NT-5$_L$, NT-4$_L$; NT-4$_R$, NT-5$_R$)=(AC; GT) and the number of BSs exhibiting this combination. Additionally, recognition degeneracies are represented by three types of arrows, i.e., —> (asymmetrical intrinsic), <—> (symmetrical intrinsic), and >—< (extrinsic), with an associated number giving the frequency of the non-palindromic nucleotide combination that results from the mixture of the two semisequences at the associated nodes. We highlighted significant combinations in red. The number in the upper left corner on each non-empty cell is the ratio r ($0 \leq r \leq 1$) of BSs found after the removal of those incorporating nucleotides for which TFs did not appear to exhibit a special affinity. In most alignments only a few BSs were discarded; as a consequence, the value of r is usually large. Finally, cells containing an isolated node correspond to BS alignments in which only one significant (and palindromic) combination was observed. The set of recognition correlations involves a total of 811 BSs and 623 TFs. Note that the correlations presented here (NTs vs. AAs) are represented in a inverse way to those of Figure 4.A main text (AAs vs. NTs). See Text S1 for a more detailed explanation.

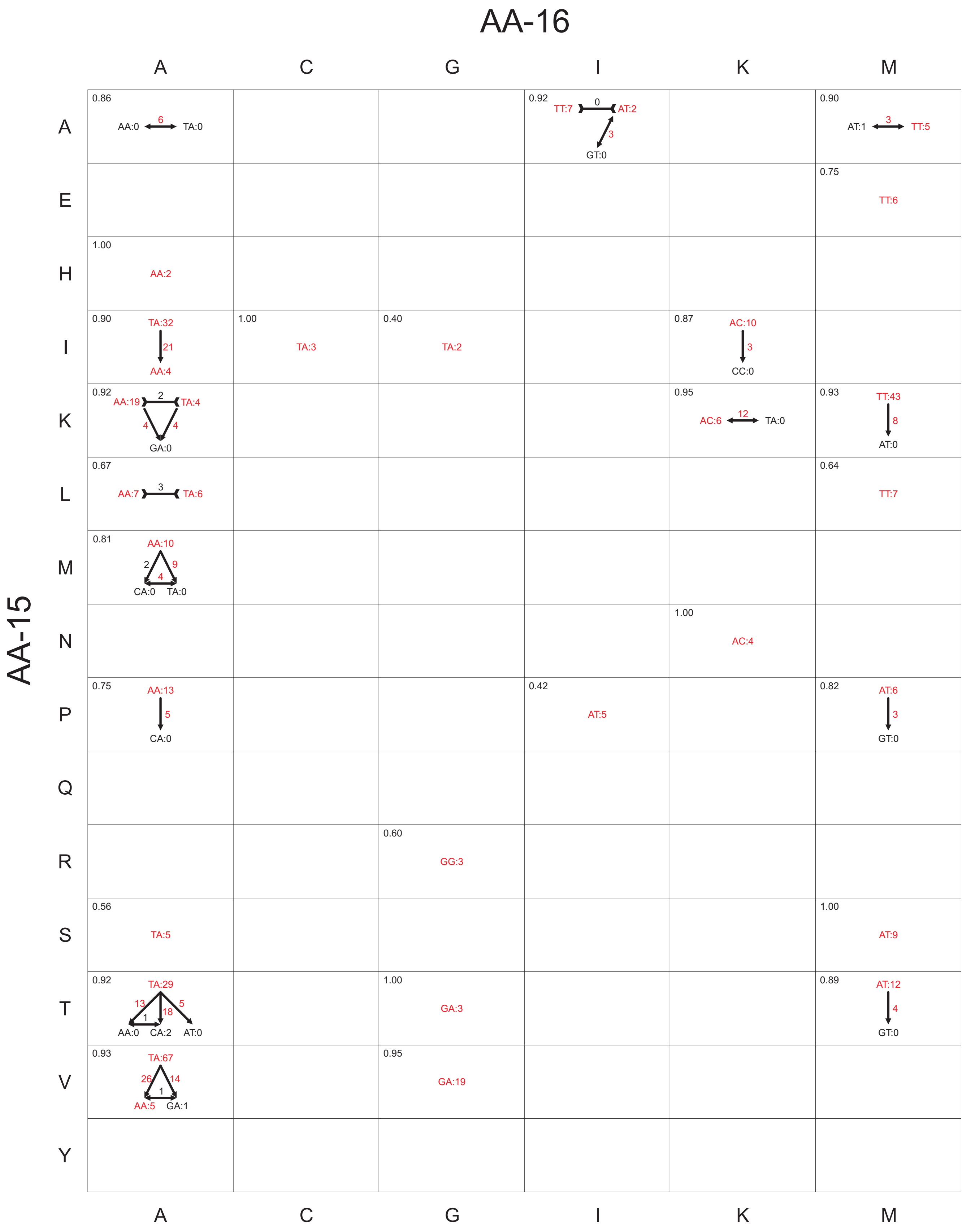

AA-16
A C G I K M
AA-15
A E H I K L M N P Q R S T V Y
0.86 AA:0 6 TA:0
0.92 TT:7 0 AT:2 3 GT:0
0.90 AT:1 3 TT:5
0.75 TT:6
1.00 AA:2
0.90 TA:32 21 AA:4
1.00 TA:3
0.40 TA:2
0.87 AC:10 3 CC:0
0.92 AA:19 2 TA:4 4 4 GA:0
0.95 AC:6 12 TA:0
0.93 TT:43 8 AT:0
0.67 AA:7 3 TA:6
0.64 TT:7
0.81 AA:10 2 9 4 CA:0 TA:0
1.00 AC:4
0.75 AA:13 5 CA:0
0.42 AT:5
0.82 AT:6 3 GT:0
0.60 GG:3
0.56 TA:5
1.00 AT:9
0.92 TA:29 13 5 1 18 AA:0 CA:2 AT:0
1.00 GA:3
0.89 AT:12 4 GT:0
0.93 TA:67 26 14 1 AA:5 GA:1
0.95 GA:19
A C G I K M

AA-16

AA-15

	N	P	Q	S	T	V
A				0.89 AA:0 4 CA:4		
E				0.89 GC:8		
H			1.00 TG:3 5 TT:1	1.00 CA:2	0.75 CA:0 2 TT:1	
I	1.00 TG:3 2 3 GT:1 TA:0			0.86 AA:16 7 CA:8	0.57 AA:4	
K				0.99 GG:44 2 GA:4 4 4 2 8 AG:0 CA:0 GT:0	0.64 CA:2 0 GG:5	
L			0.60 TG:3			
M						
N						
P				0.88 CA:22 17 AA:3	0.83 AA:3 7 TA:0	
Q					1.00 AA:8	
R				0.80 CA:5 0 GG:3		0.75 GA:3
S			1.00 TT:3			
T				0.69 CA:9		
V		0.75 CT:1 2 TT:0		0.38 GT:9	0.87 AA:9 4 GA:0	
Y			1.00 TG:1 4 13 CG:0 TT:0			

N P Q S T V
