# Supplementary material for: Local Gene Regulation Details a Recognition Code within the LacI Transcriptional Factor Family
Source: PLoS Comput Biol. 2010 Nov 11;6(11):e1000989. doi: 10.1371/journal.pcbi.1000989 (PMC2978694; doi:10.1371/journal.pcbi.1000989)
Supplement: Table S2 — Natural and synthetic operators for Y15Q16 and H15Q16. (0.03 MB PDF) [file pcbi.1000989.s010.pdf]

Natural BS O1 for *E. coli* LacI				
	AAT TGTGAGC·GGATAACA ATT			

Synthetic BS: O1 with delection in NT-2_R (reference [51], main text)				
	AAT TGTGAGC·GATAACA ATT			

Palindromic synthetic BSs (reference [19], main text)		
BS	BS sequence	Binding LacI mutant
SymL	AAT TGTGAGC·GCTCACA ATT	$Y_{15}Q_{16}$ (wt), $H_{15}Q_{16}$
SymL'	AAT TGTTAGC·GCTAACA ATT	$H_{15}Q_{16}$

Natural BSs for $H_{15}Q_{16}$ (this work, all in actinobacteria)				
TF VIMSS-ID	BS sequence	position	AU/NR	species
1810447	AgT TGTGAGC·GCTAACA ATT	-86	NR	AAur
3749792	tga TGTGAcC·GtTCACA tcg	-64	NR	CMM
2169839	gga TGTGAGC·GCTCACA cTc	-77	NR	Noca
2169839	tca TGTGAaC·GCTCACA ATT	-105	NR	Noca
2004131	ggT TGTTAGC·GCTAACt gaa	-65	AU	BAD
2004131	AAT aGTGAGC·GtTAACA gTg	-59	NR	BAD
2004131	AAc aGTGAGC·GCTAACA tTc	-64	NR	BAD
2268667	tAg TGTGAGC·GCTAACg caa	-41	AU	Blon
2268667	AAT TGTGAGC·GCTAACA ccc	-70	NR	Blon

Natural SymL-like BSs for $Y_{15}Q_{16}$ (this work, PF scan)				
3571242	tAg TGTGAcC·GaTCACA gca	-168	AU	Rcas (cloroflexi)
3654531	tgc TGTGAaC·GtTCACg tga	-39	NR	SACE (actino.)
3760850	gAT TGTGAGC·GtTCACA tgg	-124	AU	sce (δ-proteo.)

Table S 2: Natural and synthetic binding sites (BSs) for $Y_{15}Q_{16}$ and $H_{15}Q_{16}$. A dot distinguishes the half sites. Colors identify different palindromic or mixed combinations in the quartet (NT-5_L, NT-4_L; NT-4_R, NT-5_R). Flanking nucleotides are separated by a space to help visualization of the highly conserved central region of the BSs. Positions are relatives to the start codon of the first downstream regulated gen. The intergenic regions in which the BSs were found (see Figure 2.A, main text) are indicated by AU (autoregulation) or NR (downstream neighbor regulation). Natural BSs for the recognition sequence $H_{15}Q_{16}$ are very similar to either the perfect palindromic sequences of SymL and SymL', or their mixture. For $Y_{15}Q_{16}$ we also found several BSs in the first raw phylogenetic footprinting (PF) scan that resemble the synthetic SymL construction. Species abbrev.: AAur, *Arthrobacter aurescens TC1*; BAD, *Bifidobacterium adolescentis ATCC 15703*; Noca, *Nocardioides sp. JS614*; Blon, *Bifidobacterium longum DJO10A*; CMM, *Clavibacter michiganensis subsp. michiganensis NCPPB 382*; Rcas, *Roseiflexus castenholzii DSM 13941*; SACE, *Saccharopolyspora erythraea NRRL 2338*; sce, *Sorangium cellulosum 'So ce 56'*.
