# Supplementary material for: Local Gene Regulation Details a Recognition Code within the LacI Transcriptional Factor Family
Source: PLoS Comput Biol. 2010 Nov 11;6(11):e1000989. doi: 10.1371/journal.pcbi.1000989 (PMC2978694; doi:10.1371/journal.pcbi.1000989)
Supplement: Table S3 — Examples of two-strand-detailed binding sites. (0.02 MB PDF) [file pcbi.1000989.s011.pdf]

BS #1
5’-tgGTagc·gctACca-3’
3’-acCAtcg·cgaTGgt-5’

BS #2
5’-tgAGagc·gctCTca-3’
3’-acTCtcg·cgaGAgt-5’

BS #3
5’-tgGTagc·gctCTca-3’
3’-acCAtcg·cgaGAgt-5’

BS #4
5’-tgAGagc·gctACca-3’
3’-acTCtcg·cgaTGgt-5’

Table S3: Examples of two-strand-detailed binding sites (BSs). A dot distinguishes the half sites. #1 and #2 are palindromes. Non-palindromic BSs #3 and #4, built from mixed combinations of the palindromes’ half sites, are identical excepting orientation. NT-4 and NT-5 positions are colored to help visualization (compare with Figure 3.A, main text). The rest of positions arbitrarily displays the nucleotides of SymL (see Figure 4.C in main text and Table S2).
