# Supplementary material for: Local Gene Regulation Details a Recognition Code within the LacI Transcriptional Factor Family
Source: PLoS Comput Biol. 2010 Nov 11;6(11):e1000989. doi: 10.1371/journal.pcbi.1000989 (PMC2978694; doi:10.1371/journal.pcbi.1000989)
Supplement: Text S1 — Efficiency of the BS search method based on local regulation. Resolution of the recognition correlations. Comparison with mutational data. Glossary. Appendix: BS logos. (1.45 MB PDF) [file pcbi.1000989.s012.pdf]

Local Gene Regulation Details a Recognition Code for the LacI Transcriptional Factor Family

Text S1

Francisco M. Camas[1,*], Eric J. Alm[2], Juan F. Poyatos[1]

[1]Logic of Genomic Systems Laboratory, Spanish National Biotechnology Centre, Consejo Superior de Investigaciones Científicas (CSIC), Madrid, Spain
[2]Department of Biological Engineering, Massachusetts Institute of Technology, Cambridge, MA, USA
*To whom correspondence should be addressed; E-mail: fmcamas@gmail.com

Abbrev.: BS, binding site; HTH, helix-turn-helix; PF, phylogenetic footprinting; PWM, position weight matrix; RTB, RegTransBase; TF, transcriptional factor.

Contents

1 Efficiency of the BS search method based on local regulation 2

2 Resolution of the recognition correlations 3
- 2.1 Notation criteria . . . 3
- 2.2 Significant states . . . 4
- 2.3 Extrinsic *vs.* intrinsic degeneracies . . . 6
- 2.4 Symmetrical *vs.* asymmetrical degeneracies . . . 7
- 2.5 Integration of all scenarios for degeneracy . . . 8

3 Comparison with Milk *et al.* mutational data 9

Glossary 11

References 13

Appendix: BS logos 15

1 Efficiency of the BS search method based on local regulation

RegTransBase (RTB) is an extensive database of prokaryotic binding sites (BSs)[1]. BSs are grouped in alignments associated to ortholog transcriptional factors (TFs). These alignments are based on experimental and/or bioinformatic approaches. For each alignment, the sets of TFs and BSs are displayed separately and there may be several TFs corresponding to a same organism. Most alignments are built from BSs recognized by LacI family members. In this case, orthologs associated to a same alignment could differ in their respective recognition helices. Thus, to compare our results with RTB data (contemporary version v5) we proceed as follows:

i) within each alignment, and whenever it was possible in an univocal way, we assigned as potential regulator of a given BS the TF that is encoded in the same genome;

ii) we removed those TFs with redundant HTH-LacI domains (using the same criterion we applied over our own data);

iii) redundancies associated to double annotation of BSs regulating divergent operons were also removed;

iv) BSs were regrouped in sets, each associated to a different recognition helix.

The advantages of our BS search method based on local regulation lie in: i) the chance of its direct application over each annotated genome and ii) the avoidance of those problems related to orthology and functionality –it is trivial that in the case of autoregulation the functional relationship between the TF and the regulated operon does exist. On the other hand, the main disadvantage of this method when compared with orthology-based ones is the exclusion as a target of search of those BSs which are not located in the neighborhood of the gene encoding the TF. Thus, the number of BSs that the local approach can relate to a same domain will be smaller on average.

However, as our search spans over every sequenced and annotated genome, we associated BSs to a large number of TFs, what was vital to define the level of universality of our conclusions. The number of TFs with at least a found BS –712 out of 1490, involving 572 autoregulations and 207 downstream neighbor regulations (see Figure 2.A, main text)– almost triplicates the 271 members of the TVSR set[1] in RTB v5 (Figure S5.A), after the process above was applied. Moreover, the big number of considered TFs compensates the local limitation of the search because the total number of found BSs is also larger (942 vs 721 in RTB, Figure S5.B).

[1]See the Glossary section of this document.

2 Resolution of the recognition correlations

This section explains the protocol we followed to build Table S1, that contains the correlations between the pair of recognition amino acids (AA-15, AA-16) and the recognized sequences in (NT-5, NT-4). The protocol firstly removes those BSs with potential spurious nucleotides exhibiting no special affinity and next solves the different degeneracies (which are explicitely annotated in Table S1). We exemplify this method in the set of BSs associated to the recognition amino acids $K_{15}S_{16}$ because in this particularly complex case we found the three types of scenarios for degeneracy.

However, we have to introduce, first of all, the notation criteria we follow in this document for the set of nucleotides in the specificity-associated positions NT-4 and NT-5.

2.1 Notation criteria

Table S3 displays four double-stranded BS sequences. In each case, upper strand is the sense strand (where the BSs we found are always read). Let's consider now the non-palindromic sequence #3. The direct reading of the quartet of nucleotides occupying the specificity positions in both half sites is written between parentheses:

$$(\text{NT-5}_L,\ \text{NT-4}_L;\ \text{NT-4}_R,\ \text{NT-5}_R) \rightarrow (\text{GT, CT}).$$

It will be convenient to notate the positions corresponding to the right *semisequence* (see Glossary) as read from 5' to 3' in the antisense strand. In this case, we use square brackets (c for complementary nucleotides):

$$[\text{NT-5}_L,\ \text{NT-4}_L;\ \text{NT-5}^c_R,\ \text{NT-4}^c_R] \rightarrow [\text{GT, AG}].$$

Palindromes –as sequences #1 y #2 in Table S3– can be easily identified under this last notation:

$$(\text{GT, AC}) \rightarrow [\text{GT, GT}] \qquad (\text{AG, CT}) \rightarrow [\text{AG, AG}].$$

There are $4^4 = 256$ potential combinations of this type. The frequency $\tilde{f}$ in which each of these combinations appears in the BS alignment associated to a same amino-acid recognition sequence can be arranged in a matrix of elements $\tilde{f}_{i,j}$, where both subindexes run over the 16 possible combinations of two nucleotides on each semisequence. The sum of all the frequencies in the matrix is the number N of BSs in the alignment. Under the square-bracket notation, frequencies corresponding to palindromes are located in the main diagonal, $\tilde{f}_{i,i}$; and non-palindromic combinations which are invariants excepting orientation interchange their row and column indexes ($\tilde{f}_{i,j} \leftrightarrow \tilde{f}_{j,i}$). This is the case of those combinations extracted from BSs #3 and #4 in Table S3: [GT, AG] and [AG, GT], respectively.

In fact, we consider as equivalents –in what refers to the binding energy– those sequences exhibiting this mere orientation difference[2]. Thus, we group them in what we define as a same *state*, which is notated between curly brackets:

$$\{\mathrm{AG, GT}\}=[\mathrm{AG, GT}] \cup [\mathrm{GT, AG}]=(\mathrm{AG, AC}) \cup (\mathrm{GT, CT}).$$

For palindromic cases there is only a semisequence combination per state. We can also abbreviate the notation for palindromes without lost of information:

$$\{\mathrm{AG}\}=[\mathrm{AG, AG}]=(\mathrm{AG, CT}).$$

This is the final notation for our space of semisequence combinations, which is now restricted to $16 \times (16 + 1)/2 = 136$ different states. Consequently, the set of frequencies for all the states can be arranged in a triangular matrix (including the main diagonal for palindromes). This matrix (called F) is related with the previous 16 x 16 one through $f_{i,j} = \tilde{f}_{i,j} + \tilde{f}_{j,i}$ for non-palindromic states and $f_{i,i} = \tilde{f}_{i,i}$ for palindromes. The sum of all the frequencies in the matrix is again the number N of BSs associated to a same recognition sequence.

Figure S6 shows the matrix F corresponding to $K_{15}S_{16}$. The most populated state is the palindrome {GG} with 44 instances. Coming back to the most conventional notation, this dominant palindrome (GG, CC) is reflected in the biggest letters exhibited by the logo (Figure S6, insert) in the quartet of positions (NT-5_L, NT-4_L; NT-4_R, NT-5_R).

2.2 Significant states

A given BS could incorporate nucleotides for which the corresponding TF does not have a special affinity –although the BS could be still functional. The incorporation of such low-affinity nucleotides in a degenerate position (as it is often the case of NT-4 and NT-5) has a small penalty under a PWM-based BS search –in contrast to the big penalties associated to the substitution of a clearly dominant nucleotide. As we were interested only in those nucleotides for which the corresponding TF exhibited at least a minimal moderate affinity, we removed all those semisequences without a minimal statistical significance when compared with a null model in which they arise as neutral combinations of the genomic background.

As a first step, we identified those states whose frequencies are significantly large when compared with a null model in which the probability of a state is simply determined by the probability of the corresponding nucleotide sequences in the intergenic background. That is, and following with our previous examples, the probability in the null model of the states {AG; GT} and {AG} would be

[2] Although in our example the invariance between #3 and #4 involves every BS coordinate, we only require the specificity positions to satisfy this invariance: [GT, AG]∼[AG, GT].

given by

$$\begin{aligned} p_{\{AG;GT\}} &= p_{[AG;GT]} + p_{[GT;AG]} = p_{(AG;AC)} + p_{(GT;CT)} = \\ &= p_A\, p_G\, p_A\, p_C + p_G\, p_T\, p_C\, p_T, \\ p_{\{AG\}} &= p_{[AG;AG]} = p_{(AG;CT)} = p_A\, p_G\, p_C\, p_T, \end{aligned}$$

respectively, being p_i ($i = A, C, G, T$) the background probability of the four nucleotides, which is calculated from the nucleotide content of the sense strand[3]. To compute the p-value of each state, we built a set of 10^5 random F-matrices, $F^{(rnd)}$, in which the population of each of the 136 states is determined by its null-probability, $p_{\{s\}}$ ($s = 1 \ldots 136$). Being the single nucleotide probability normalized ($\sum_{i=A,C,G,T} p_i = 1$), so are the $p_{\{s\}}$'s.

Algorithm S1 Pseudocode for the $F^{(rnd)}$ matrices set generation.

```
for j = 1 to 99999 do
  for i = 1 to N do
    generate u according to U(0|1)
    select s such that c_s = max({c_r | c_r ≤ u})
    f_{s,j}^(rnd) ← f_{s,j}^(rnd) + 1
  end for
end for
```

The set of matrices $F^{(rnd)}$ was built following Algorithm S1 where the quantities c_s ($s = 1 \ldots 136$) are the starting points in which the unitary segment [0, 1] is divided, so that each partition length equals the probability of one state:

$$c_s = \begin{cases} 0 & s = 1, \\ \sum_{r=1}^{s-1} p_{\{r\}} & s = 2 \ldots 136. \end{cases}$$

The inner loop of Algorithm S1 generates N times a random number u following the standard uniform distribution $U(0,1)$. The value of each generated u selects one state s whose frequency increases in one unity. In the outer loop 99999 trials[2] generate the same number of matrices $F^{(rnd)}$. Finally, for each of the 136 states s we calculated the p-value P_s associated to the observed frequencies f_s when compared with the set of random frequencies associated to the same state $f_{s,j}^{(rnd)}$ ($j = 1 \ldots 99999$),

$$P_s = \frac{(\text{number of } f_{s,j}^{(rnd)} \text{ for which } f_{s,j}^{(rnd)} \geq f_s) + 1}{10^5}.$$

In Figure S6 significantly large frequencies ($P_s < 0.05$ after correcting for multiple testing[3]) are highlighted in red. Finally, we discarded those semisequences

[3]Specifically, the random probability of each base was calculated from its frequency in the set of intergenic regions of search associated to the corresponding recognition class.

that were not involved in any significant state. When the corresponding rows and arrows were removed from matrix F we obtained a triangular submatrix. We named this submatrix as matrix S. Figure S7.A shows that extracted from Figure S6. Note that, aside all the significant frequencies (that inherit red color from the original matrix F) several non-significant ones are included in S. The previous protocol was applied over each set of BSs associated to a same sequence of recognition amino acids, i.e., to a same recognition TF class[4].

Once we obtained matrix S, we solved the different degeneracy scenarios (see Figure 3.B, main text). We first separated intrinsic from extrinsic degeneracies. A second step distinguished, inside the intrinsic scenario, between symmetrical and asymmetrical recognitions. We detail this next.

2.3 Extrinsic *vs.* intrinsic degeneracies

Let's consider the matrix S for $K_{15}S_{16}$ in Figure S7.A[5]. The palindromic states P1={GA} and P2={GG} are significant. However, this is not the case of the corresponding mixture M={GA, GG} (circled in blue). This suggests a scenario of extrinsic degeneracy between the sets of TFs binding P1 and P2, respectively (see main text, Figure 3.B, right). In general, a matrix S can involve more than two palindromic states and, in consequence, more than one mixture –for example, matrix S in Figure S7.A involves 5 different semisequences that can be combined in 5 palindromic states and 10 potential mixtures. Thus, when the matrix S involves more than two different semisequences we detected significantly low-frequency mixtures by contrasting the extant frequencies in S with a null model in which semisequences combine randomly (this penalizes underrepresented mixtures).

The null model was built as follows: i) we counted the frequency of each semisequence in matrix S, taking into account that each palindromic case contributes with two semisequences of the same type and each mixture adds one instance of two different semisequences –for example, there are $44 \times 2 + 2 + 4 + 4 = 98$ semisequences GG and $4 \times 2 + 2 + 8 = 18$ semisequences GA in Figure S7.A, ii) the probability of a given semisequence equals the number of this type of semisequence divided by the total number of semisequences (2 x n) in S, being n the total sum of frequencies in this matrix ($n = 68$ ergo 136 semisequence counts in Figure S7.A), iii) the probability of a semisequence combination is given by the product of the probabilities of its constituting semisequences, and iv) the probability of a state is given by the sum of the probabilities of its corresponding combinations (recall that a non-palindromic state contains two combinations).

Finally, we made 10^4 random trials with an algorithm similar to Algorithm S1, substituting N for n and using the state probabilities. Each mixture with

[4]We did not detect any significant frequency in the F matrix associated to $H_{15}T_{16}$. In this exceptional case, we selected as significant the state corresponding to the largest frequency.

[5]Note how it is built from the cells highlighted in dark gray in Figure S6.

a significant low frequency ($P < 0.05$ after correcting for multiple testing) was associated to an event of extrinsic degeneracy. For instance, the circled state in Figure S7.A was confirmed as such a critical mixture. In this case, the extrinsic degeneracy would imply that there exists a subset of regulators of the $K_{15}S_{16}$-class that is able to bind semisequence GA but not GG, and other subset with the opposite behavior[6]. In principle, we did not exclude that both groups can share affinities for third semisequences.

Thus, in the presence of a low-frequency mixture we extracted two submatrices from matrix S[7]. Each submatrix was obtained by removing all those combinations having one of the semisequences of the mixture as constituent. This can be easily view if we redefine the matrix indexes associated to each semisequence of S in such a way that the critical combination is now placed in the upper right corner of the matrix (Figure S7.B). The submatrices are then extracted by removing alternatively the first row and the last column of the reordered S matrix[8]. Finally, within each submatrix we removed rows and columns corresponding to semisequences only involved in zero-frequency combinations (as in the case of the upper submatrix in Figure S7.C).

2.4 Symmetrical *vs.* asymmetrical degeneracies

Except in the trivial case of 1 x 1 submatrices, the different semisequences in the submatrices were associated to an intrinsically degenerate recognition. Next, we wanted to determine its symmetrical (Figure 3.B, left) or asymmetrical (Figure 3.B, center) character. We assumed within each submatrix (or in matrix S when no events of extrinsic degeneracy were detected) a starting null model of intrinsic *symmetrical* recognition, in which all the TFs have similar affinities for each combination of the considered semisequences. If all these combinations suffer the same selective pressure as BSs constituents, we could expect that, after the selection, the distribution of the respective frequencies is only determined by the conditional probability that results from the restriction (and subsequent renormalization) of the neutral genomic-background probability we used in Section 2 to the combinations considered in the given submatrix. For example, in this symmetrical null model the post-selection probability p' of each state in the upper submatrix of Figure S7.C would be

$$
\begin{aligned}
p'_{\{GA\}} &= \frac{1}{norm} p_{\{GA\}}, \\
p'_{\{GA,GT\}} &= \frac{1}{norm} p_{\{GA,GT\}},
\end{aligned}
$$

[6]This result could be questioned if at least two different members of the triad [P1, M, P2] were associated to a same TF. We never found such a case.

[7]In absence of such a mixture, we jumped directly to the next step of the protocol.

[8]All this process complicates if more than one critical mixture were found per set of BSs. In practice, we did not encounter this case.

$$p'_{\{GT\}} = \frac{1}{norm} p_{\{GT\}},$$

$$\text{with } norm = p_{\{GA\}} + p_{\{GA,GT\}} + p_{\{GT\}}.$$

Next, we compared the extant frequencies in the submatrix with a set of 10^4 random submatrices where the frequencies are distributed under this probability. Randomizations were made with an algorithm similar to Algorithm S1, substituting N for the sum of all frequencies in the submatrix and using the p' probabilities. When a palindrome is found with particularly high frequency (being statistically significant with $P < 0.05$ after correcting for multiple testing) the submatrix was associated to a scenario of asymmetrical intrinsic recognition, where this palindrome dominates (as P1 in Figure 3.B, center). In absence of such large frequencies or when they were associated to a mixture, we kept the symmetrical situation of the null model.

2.5 Integration of all scenarios for degeneracy

The upper matrix in Figure S7.C lacks a dominant palindrome. This is thus a case of intrinsic symmetrical degeneracy. The corresponding graph in Figure S7.D schematizes this relationship by means of a bidirectional arrow connecting the palindromic states. The frequency and semisequence of the palindromes are placed at the extremes of the arrow and the number at the middle of the arrow is the frequency of the mixed state. We kept the original red color for significant frequencies. On the contrary, in the lower submatrix of Figure S7.C the (circled in green) palindrome {GG} dominates. In this case we used unidirectional arrows directed from the dominant to the dominated palindromes (Figure S7.D). We did not plot arrows for zero-frequency mixtures.

Figure S7.E integrates all the scenarios of degeneracy we found: the two graphs in Figure S7.D are joined and the extrinsic relationship in Figure S7.B is added (using special double-head arrows). This resultant graph, which covers all the n frequencies in matrix S, is finally included in the Table of Correlations (Table S1).

3 Comparison with Milk *et al.* mutational data

The agreement of our theoretical predictions with mutational data seemed to be in conflict with the following conclusion of the mutational study by Milk *et al.*: comparative genomics is not sufficient for predicting synthetic (AA-15, AA-16, AA-20)/(NT-6, NT-5, NT-4) associations[4]. This followed from an experiment in which the sequence of the recognition triplet (AA-15, AA-16, AA-20) of LacI was swapped for that of MalR, RbtR, FruR, PurR, RbsR, GalR, CytR, RafR and ScrR (the last four belonging to the TVSR set). For each of these regulators the sequence of (NT-6, NT-5, NT-4) in the symmetrical operator SymL was changed to that identified in one of the natural BSs the regulator binds. Only the mutants associated to the recognition sequence of GalR and FruR were able to bind the corresponding operator[4].

The nucleotides occupying the specificity associated positions in a particular BS are not always those for which the TF has the strongest preference. The rest of protein/DNA contacts could suffice for a functional binding (note how a large fraction of the mutants in Figure S3 are still able to bind with the wild type sequence of SymL). To avoid this effect, the binding preferences of a given recognition sequence should be better identified through the PWM (or its logo) for the corresponding BS alignment[5]. Thus, it could be that the use of single BSs could be influencing the mentioned conclusion. To investigate this, we contrasted the nucleotide sequences that were tested in the mutational work with those predicted by our PWM-based recognition associations. For the five TFs which do not belong to the TVSR set, the sequences of the BSs used in the mutational work correspond in general to bases with a high information content in the respective logo (data not shown). This confirmed then the unpredictability conclusion for this small set of non–TVSR members of the LacI family.

However, Figure S3 suggested that a different scenario could be applying within the TVSR set. Moreover, the existence of highly conserved positions in the consensus logo of Figure 2.E implies that for many TFs in this set there is possibly a canonical mode of binding associated to an ideal BS backbone given by the sequence (T)G--A-CG-T--C(A). In this case, we could reasonably expect that mutational experiments on the (AA-15, AA-16) pair would recover the predicted new specificities on the (NT-5, NT-4) positions. This is the case of the LacI mutant with the recognition sequence of GalR (AA-15, AA-16)=VA, which is able to bind the SymL variant with (NT-5, NT-4)=TA[4, 6, 7]. This is also in agreement with our genomically-derived predictions (Figure 4.A, main text). On the contrary, the chance for successful experimental redesign would be lower when the reference BSs diverge significantly from the consensus pattern. In fact, the exceptions to the dominant mode of binding within the TVSR set includes the regulators CytR, RafR and ScrR. This exceptionalness explains why the LacI mutants with the recognition sequence of these regulators fail to bind SymL variants

built from their respective natural BSs.

The case of CytR is the most clear case of a missed positive result in the mutational test. For the corresponding SymL variant, Milk *et al.* used the (NT-6, NT-5, NT-4) sequence of a BS from which *Escherichia coli*'s CytR regulates the transcription of the *deo* operon. It is known that this particular regulator exhibits an exceptional mode of binding within the LacI family. Effectively, *E. coli*'s CytR does not use hinge-helix for minor groove recognition[8] and needs to constitute a regulatory complex with CRP to bind the DNA[9]. However, our results for the TF recognition class with the same recognition sequence of CytR, (AA-15, AA-16)=TA, showed that a more canonical behavior dominates in this class. As reflected in Figure 4.A, this pair recognizes (NT-5, NT-4)=TA preferably. Accordingly, a SymL variant with this sequence was associated to $T_{15}A_{16}$ in the mutational screening (Figure S3).

Since RafR and ScrR shares the same recognition sequence $V_{15}T_{16}$, both regulators were associated to a same LacI mutant. This mutant was tested against SymL variants also designed from two natural BSs which did not respond to the consensus behavior of Figure 2.E. Both of these BSs lacked the conserved guanine in NT-6. In fact, the arginine in AA-20 of the close orthologs of ScrR (like SacR in *Lactobacillus plantarum*) possibly contacts a guanine in NT-7 (instead of NT-6) giving wider BSs[10]. See the section of conclusions in the main text for comments on the loss of non-canonical BSs in our search protocol.

Glossary

Half site: The BSs for the LacI family exhibit a palindromic or almost palindromic nature inherited from the axial symmetry of the binding dimers (see Figure 1.A, main text). A half site is constituted by the sequence of nucleotides to the left or right side of the BS center. The sequences of both half sites can be directly compared by reading the right half site in the 5'-to-3' sense over the complementary DNA strand.

TVSR set: The majority set of members of the LacI family of domains exhibiting the TVSR sequence of AAs in the range of recognition helix positions from AA-17 to AA-20 (see Figure S2). Our analysis is restricted to this leading subgroup.

AA-15 and AA-16: The main specificity-associated positions in the helix-turn-helix (HTH) recognition domain. We refer to the coordinates of any amino acid in a particular HTH-LacI domain by the position (plus the prefix AA-) that the given amino acid has in the domain alignment of Figure 1.B, main text.

Recognition class: TFs of the TVSR set sharing the same (AA-15, AA-16) recognition sequence.

NT-4 and NT-5: The main specificity-associated positions in each half site –four nucleotide positions in total, but usually cited as a pair (NT-5, NT-4), see next entry.

Semisequence: In this work we restrict the term *semisequence* for the left or right components of the quartet (NT-5_L, NT-4_L; NT-4_R, NT-5_R). Under our notation criteria, semisequences are always read from 5' to 3' and cited as a pair (NT-5, NT-4). In this way, palindromic combinations for this quartet are easily identified, and so are the relationships between mixtures and palindromes. When we refer to a full BS, we use the term *half site* instead of *semisequence* to avoid confusion.

Combination (of semisequences): We often use this term to refer the sequence (NT-5_L, NT-4_L; NT-4_R, NT-5_R) because it can be formally understood as an assembly of its constituting semisequences. This also inspires our matrix-based approach. There are $4^4 = 256$ possible combinations.

State: We group two complementary combinations in a same state because we consider that they are equivalent in what refers to specificity. Thus, there are 136 possible states.

Palindrome: In general, it is a nucleotide sequence equals to its complementary one (both read in the same 5'-to-3' or 3'-to-5' sense). For instance, this is the case of synthetic palindromic BSs as SymL: `5'-AATTGTGAGC·GCTCACAATT-3'`. However, in most cases we restrict this term for the specificity-associated quartet (NT-5_L, NT-4_L; NT-4_R, NT-5_R). Thus, we talk about palindromes when (NT-5_L, NT-4_L) = (NT-5^c_R, NT-4^c_R), with c for complementary nucleotides.

Mixture: We apply this term to each given non-palindromic state because this can be formally understood as the assembly of the semisequences of two different palindromes. For example, the mixture M={GA, GG} is built from the semisequences of palindromes P1={GA} and P2={GG}. Note how our notation criteria enhances this formal approach.

Matrix F: Triangular matrix containing the frequencies of all the 136 states in the BS set associated to a same *recognition class*. See an example in Figure S6. Due to our notation criteria, palindromic combinations are easily located in the main diagonal.

Matrix S: Triangular submatrix extracted from matrix F. Only semisequences involved in at least a significant state are considered. Palindromic combinations in main diagonal.

Specificity degeneracy: In a broad sense, it is the ambiguity exhibited by a BS-logo when two or more nucleotides compete for any of the specificity-associated positions.

Intrinsic degeneracy: The specificity degeneracy has an intrinsic nature if the set of TFs sharing the same recognition amino acids is able to bind the competing nucleotides. Thus, each TF exhibit a (same) degenerate specificity, strictly speaking. See Figure 3.B (left and center) in main text.

Extrinsic degeneracy: In contrast to intrinsic ones, extrinsic degeneracies do not involve a truly recognition degeneracy for each isolated TF. In this case the ambiguity exhibited by the logo is due to the alignment of BSs recognized by TFs with different specificities despite their shared (AA-15, AA-16) sequence. See Figure 3.B (right) in main text.

References

[1] Kazakov, A. E., Cipriano, M. J., Novichkov, P. S., Minovitsky, S., Vinogradov, D. V., Arkin, A., Mironov, A. A., Gelfand, M. S., and Dubchak, I. (2007) RegTransBase - a database of regulatory sequences and interactions in a wide range of prokaryotic genomes. *Nucleic Acids Res.*, **35**, D407–D412.

[2] Noreen, E. W. (1989) *Computer-Intensive Methods for Testing Hypotheses: An Introduction*. Wiley-Interscience.

[3] Benjamini, Y. and Hochberg, Y. (1995) Controlling the false discovery rate - a practical and powerful approach to multiple testing. *J. Royal Statistical Soc. Series B-methodological*, **57**, 289–300.

[4] Milk, L., Daber, R., and Lewis, M. (2010) Functional rules for lac repressor-operator associations and implications for protein-DNA interactions. *Protein Science*, **19**, 1162–1172.

[5] Stormo, G. D. (2000) DNA binding sites: representation and discovery. *Bioinformatics*, **16**, 16–23.

[6] Sartorius, J., Lehming, N., Kisters, B., von Wilcken-Bergmann, B., and Müller-Hill, B. (1989) *lac* repressor mutants with double or triple exchanges in the recognition helix bind specifically to lac operator variants with multiple exchanges. *EMBO J.*, **8**, 1265–1270.

[7] Salinas, R. K., Folkers, G. E., Bonvin, A. M. J. J., Das, D., Boelen, R., and Kaptein, R. (2005) Altered specificity in DNA binding by the lac repressor: A mutant lac headpiece that mimics the gal repressor. *ChemBioChem*, **6**, 1628–1637.

[8] Jørgensen, C. I., Kallipolitis, B. H., and Valentin-Hansen, P. (1998) DNA-binding characteristics of the *Escherichia coli* CytR regulator: a relaxed spacing requirement between operator half-sites is provided by a flexible, unstructured interdomain linker. *Mol Microbiol*, **27**, 41–50.

[9] Sogaard-Andersen, L., Pedersen, H., Holst, B., and Valentin-Hansen, P. (1991) A novel function of the cAMP-CRP complex in escherichia-coli: cAMP-CRP functions as an adapter for the CytR repressor in the deo operon. *Mol. Microbiology*, **5**, 969–975.

[10] Francke, C., Kerkhoven, R., Wels, M., and Siezen, R. J. (2008) A generic approach to identify transcription factor-specific operator motifs; inferences for LacI-family mediated regulation in *Lactobacillus plantarum* WCFS1. *BMC Genomics*, **9**, 145.

[11] Crooks, G. E., Hon, G., Chandonia, J. M., and Brenner, S. E. (2004) WebLogo: a sequence logo generator. *Genome Research*, **14**, 1188–1190.

Appendix: BS logos

Each logo corresponds to the alignment of the set of BSs associated to a recognition class –constituted by TFs sharing the same (AA-15, AA-16) recognition sequence. All the considered domains exhibit the TVSR sequence in the range of recognition-helix positions from AA-17 to AA-20. Above each logo we show the amino-acid recognition sequence and a triad of numbers (i/ii/iii) corresponding to i) the total number of TFs with this recognition sequence, ii) the number of TFs for which at least one BS was found, and iii) the total number N of found BSs.

Below the logo other two triads of numbers appear. They distinguish the cases corresponding to autoregulations (AU) or downstream neighbor regulations (NR). Now, each triad (i/ii/iii) corresponds to i) the number of intergenic region of search, ii) the number of these regions for which at least one BS was found, and iii) the total number of found BSs in the regions. Note that in the case of autoregulation the value of i) equals the number of TFs because there is an intergenic region located upstream of every operon. However, as we only considered downstream neighbor regulations in which the TF and its potentially regulated operon are encoded in the same DNA strand (see Figure 2.A, main text), there is on average only an available downstream search region for half the TFs.

Logos were created with WebLogo correcting the information content overestimation that results from small samples[11] –this is reflected in smaller letter sizes for logos involving a more limited number of BSs.

VA 138/93/122

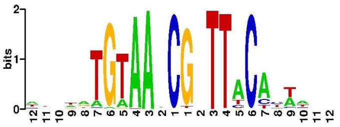

AU: 138/87/103 NR: 67/17/19

TA 101/53/74

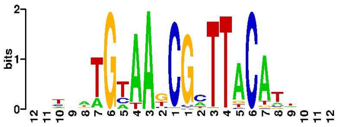

AU: 101/47/63 NR: 61/10/11

IA 91/46/63

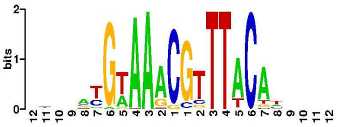

AU: 91/43/55 NR: 45/7/8

KS 87/49/69

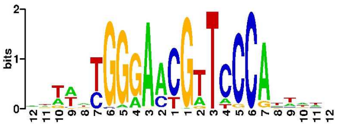

AU: 87/25/33 NR: 55/26/36

PS 76/36/48

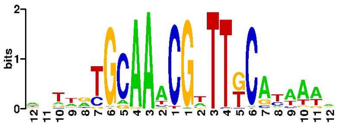

AU: 76/29/39 NR: 44/9/9

KM 68/40/55

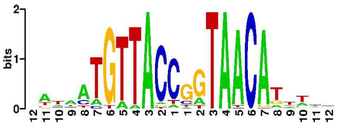

AU: 68/19/20 NR: 39/25/35

IS 57/29/36

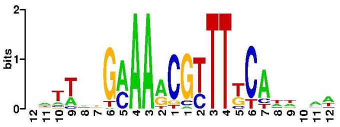

AU: 57/21/25 NR: 28/10/11

KA 50/29/36

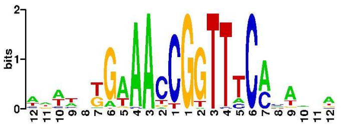

AU: 50/29/30 NR: 24/5/6

MA 47/28/31

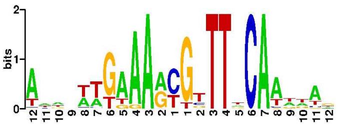

AU: 47/28/31 NR: 26/0/0

LA 44/20/24

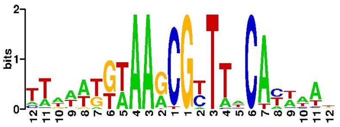

AU: 44/11/12 NR: 38/11/12

VT 40/15/15

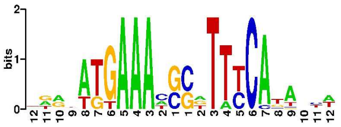

AU: 40/12/12 NR: 26/3/3

VS 36/16/24

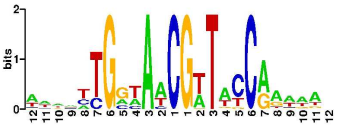

AU: 36/15/22 NR: 21/2/2

PA 34/20/24

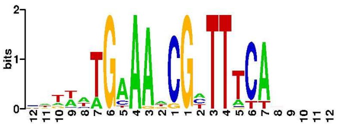

AU: 34/19/19 NR: 23/5/5

IK 29/11/15

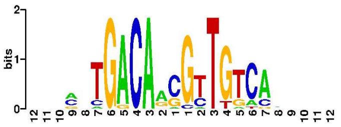

AU: 29/10/12 NR: 9/2/3

YQ 24/13/18

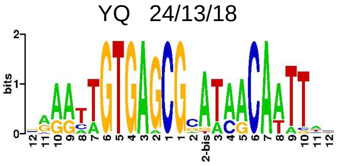

AU: 24/0/0 NR: 19/13/18

VG 24/13/20

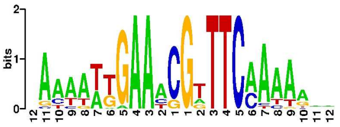

AU: 24/6/6 NR: 21/7/14

RA 22/0/0

AU: 22/0/0 NR: 8/0/0

QS 22/0/0

AU: 22/0/0 NR: 9/0/0

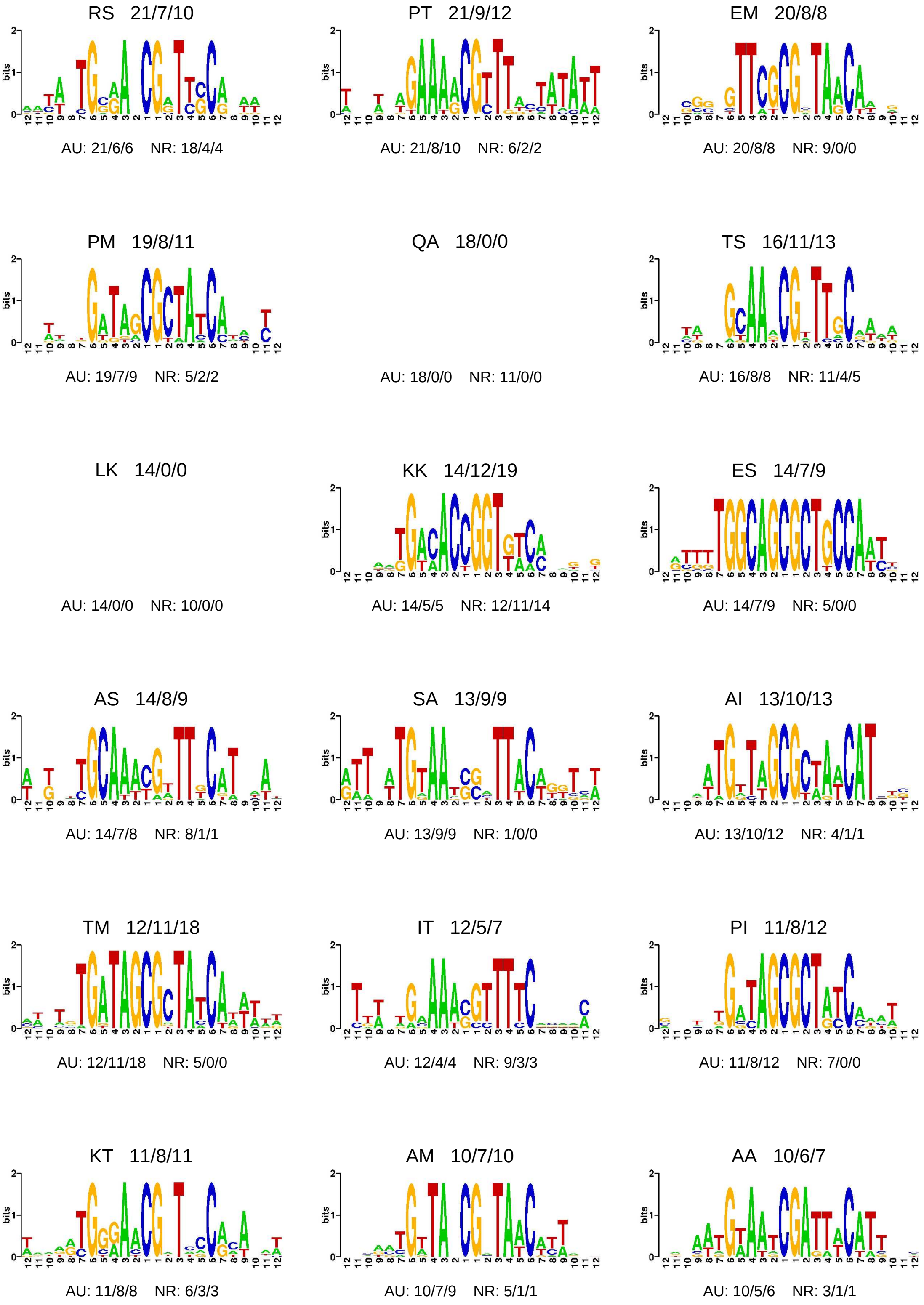
RS 21/7/10
AU: 21/6/6 NR: 18/4/4
PT 21/9/12
AU: 21/8/10 NR: 6/2/2
EM 20/8/8
AU: 20/8/8 NR: 9/0/0
PM 19/8/11
AU: 19/7/9 NR: 5/2/2
QA 18/0/0
AU: 18/0/0 NR: 11/0/0
TS 16/11/13
AU: 16/8/8 NR: 11/4/5
LK 14/0/0
AU: 14/0/0 NR: 10/0/0
KK 14/12/19
AU: 14/5/5 NR: 12/11/14
ES 14/7/9
AU: 14/7/9 NR: 5/0/0
AS 14/8/9
AU: 14/7/8 NR: 8/1/1
SA 13/9/9
AU: 13/9/9 NR: 1/0/0
AI 13/10/13
AU: 13/10/12 NR: 4/1/1
TM 12/11/18
AU: 12/11/18 NR: 5/0/0
IT 12/5/7
AU: 12/4/4 NR: 9/3/3
PI 11/8/12
AU: 11/8/12 NR: 7/0/0
KT 11/8/11
AU: 11/8/8 NR: 6/3/3
AM 10/7/10
AU: 10/7/9 NR: 5/1/1
AA 10/6/7
AU: 10/5/6 NR: 3/1/1

SM 9/6/9

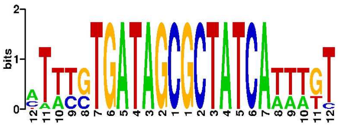

AU: 9/6/9 NR: 0/0/0

RT 9/0/0

AU: 9/0/0 NR: 5/0/0

QT 9/7/8

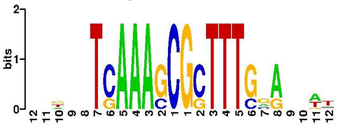

AU: 9/7/8 NR: 7/0/0

QP 9/0/0

AU: 9/0/0 NR: 4/0/0

LM 9/7/11

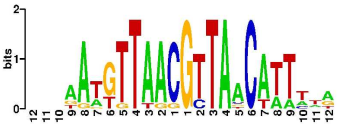

AU: 9/5/9 NR: 2/2/2

HQ 9/5/9

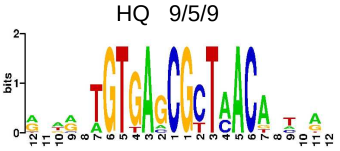

AU: 9/2/2 NR: 8/5/7

EA 9/0/0

AU: 9/0/0 NR: 8/0/0

RM 8/0/0

AU: 8/0/0 NR: 5/0/0

RG 8/5/5

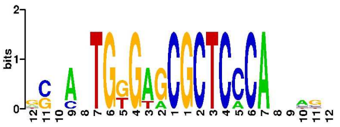

AU: 8/3/3 NR: 5/2/2

HT 8/3/4

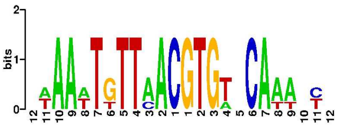

AU: 8/2/2 NR: 4/2/2

IG 7/3/5

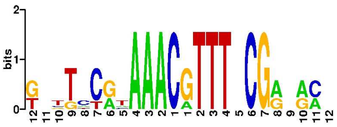

AU: 7/3/4 NR: 4/1/1

VK 6/0/0

AU: 6/0/0 NR: 4/0/0

LQ 6/5/5

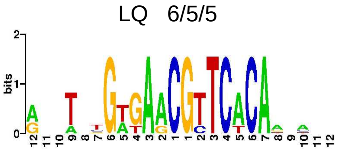

AU: 6/3/3 NR: 3/2/2

IN 6/5/9

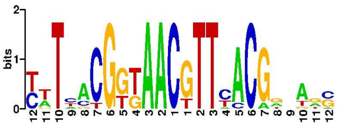

AU: 6/4/6 NR: 4/3/3

VP 5/3/4

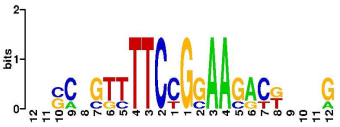

AU: 5/3/3 NR: 4/1/1

SS 5/0/0

AU: 5/0/0 NR: 2/0/0

TQ 4/0/0

AU: 4/0/0 NR: 0/0/0

TG 4/3/3

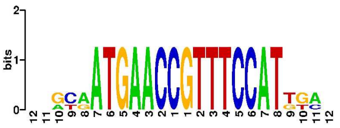

AU: 4/3/3 NR: 2/0/0

ST 4/0/0

AU: 4/0/0 NR: 3/0/0

QQ 4/0/0

AU: 4/0/0 NR: 1/0/0

PK 4/0/0

AU: 4/0/0 NR: 4/0/0

IC 4/3/3

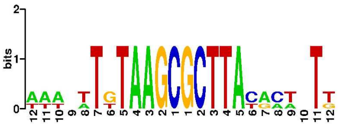

AU: 4/2/2 NR: 4/1/1

HA 4/2/2

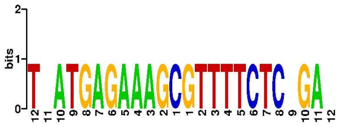

AU: 4/2/2 NR: 0/0/0

AQ 4/0/0

AU: 4/0/0 NR: 1/0/0

AG 4/0/0

AU: 4/0/0 NR: 2/0/0

YS 3/0/0

AU: 3/0/0 NR: 3/0/0

SQ 3/2/3

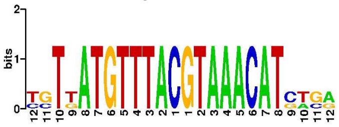

AU: 3/2/3 NR: 3/0/0

RV 3/3/4

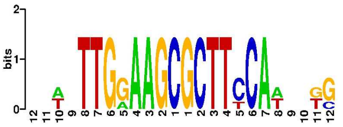

AU: 3/1/1 NR: 3/3/3

PQ 3/0/0

AU: 3/0/0 NR: 3/0/0

PG 3/0/0

AU: 3/0/0 NR: 3/0/0

NK 3/3/4

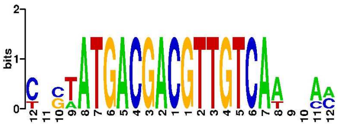

AU: 3/3/4 NR: 2/0/0

NI 3/0/0

AU: 3/0/0 NR: 0/0/0

NA 3/0/0

AU: 3/0/0 NR: 3/0/0

HS 3/2/2

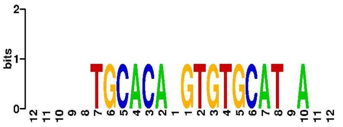

AU: 3/2/2 NR: 1/0/0

HM 3/0/0

AU: 3/0/0 NR: 2/0/0

AT 3/0/0

AU: 3/0/0 NR: 1/0/0

VN 2/0/0	TT 2/0/0	TI 2/0/0
AU: 2/0/0 NR: 2/0/0	AU: 2/0/0 NR: 1/0/0	AU: 2/0/0 NR: 0/0/0
SI 2/0/0	LT 2/0/0	LS 2/0/0
AU: 2/0/0 NR: 2/0/0	AU: 2/0/0 NR: 0/0/0	AU: 2/0/0 NR: 1/0/0
LG 2/0/0	KV 2/0/0	KI 2/0/0
AU: 2/0/0 NR: 1/0/0	AU: 2/0/0 NR: 0/0/0	AU: 2/0/0 NR: 1/0/0
KG 2/0/0	IP 2/0/0	AP 2/0/0
AU: 2/0/0 NR: 2/0/0	AU: 2/0/0 NR: 0/0/0	AU: 2/0/0 NR: 1/0/0
AK 2/0/0		
AU: 2/0/0 NR: 1/0/0		
